# Supplementary material for: Spatial transcriptomics analysis of neoadjuvant cabozantinib and nivolumab in advanced hepatocellular carcinoma identifies independent mechanisms of resistance and recurrence
Source: Genome Med. 2023 Sep 18;15:72. doi: 10.1186/s13073-023-01218-y (PMC10506285; doi:10.1186/s13073-023-01218-y)
Supplement: Supplementary file 1 — Additional file 1: Fig. S1. Heatmap of cluster markers across samples. [file 13073_2023_1218_MOESM1_ESM.pdf]

Fig. S1

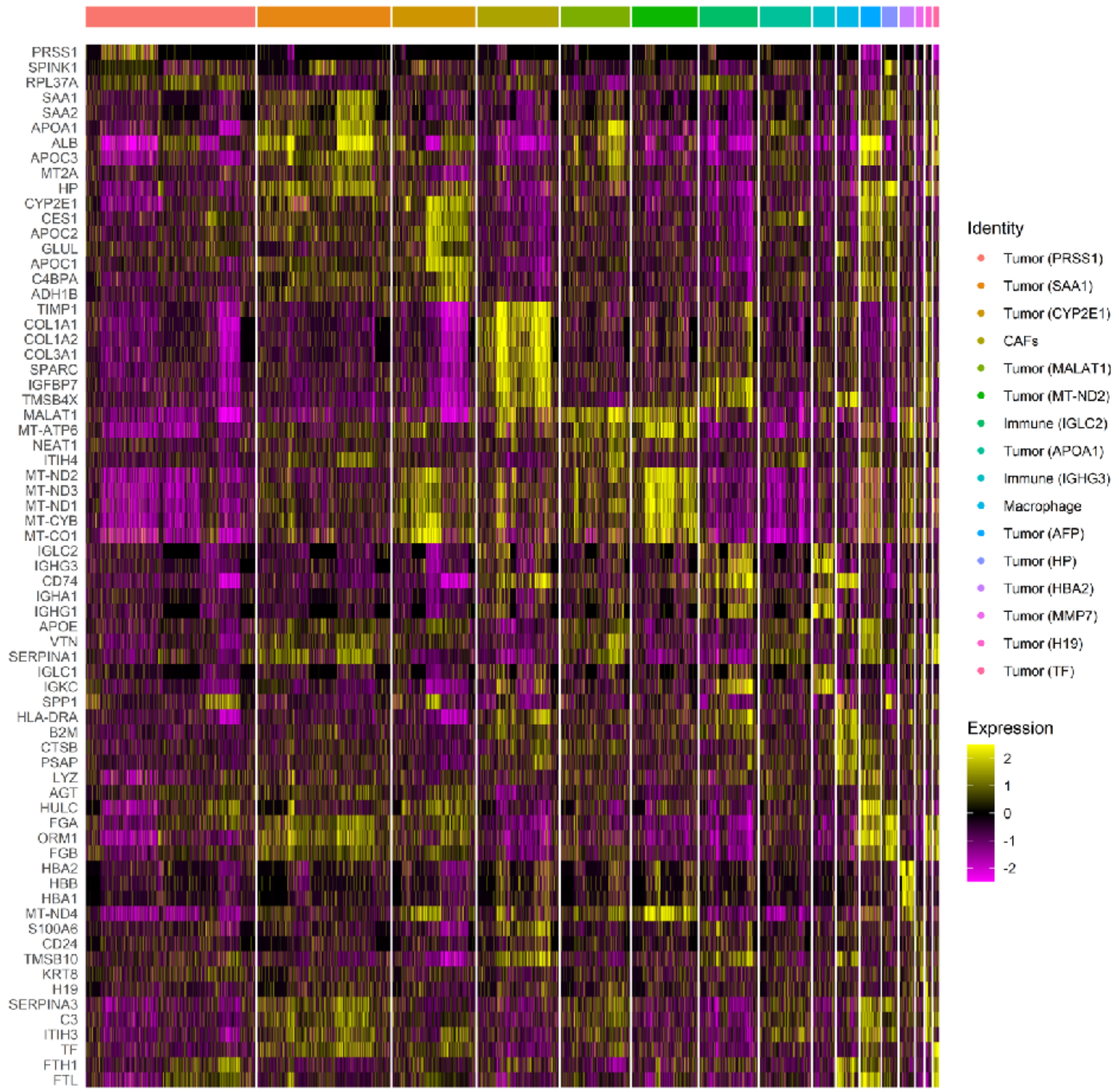

Fig. S1 - Heatmap of cluster markers across samples. Expression of highly expressed genes in each of the clusters identified in the HCC cohort. Clusters that map to the same cell type were identified by the cell type (tumor, immune or CAF) and by the highest expressed gene (in parenthesis).
