# Supplementary material for: Spatial transcriptomics analysis of neoadjuvant cabozantinib and nivolumab in advanced hepatocellular carcinoma identifies independent mechanisms of resistance and recurrence
Source: Genome Med. 2023 Sep 18;15:72. doi: 10.1186/s13073-023-01218-y (PMC10506285; doi:10.1186/s13073-023-01218-y)
Supplement: Supplementary file 3 — Additional file 3: Fig. S2. Overall expression of the antigen processing and presentation signaling pathway in the subset of spots in the spatial transcriptomics data from non-responders and responders. [file 13073_2023_1218_MOESM3_ESM.pdf]

Fig. S3

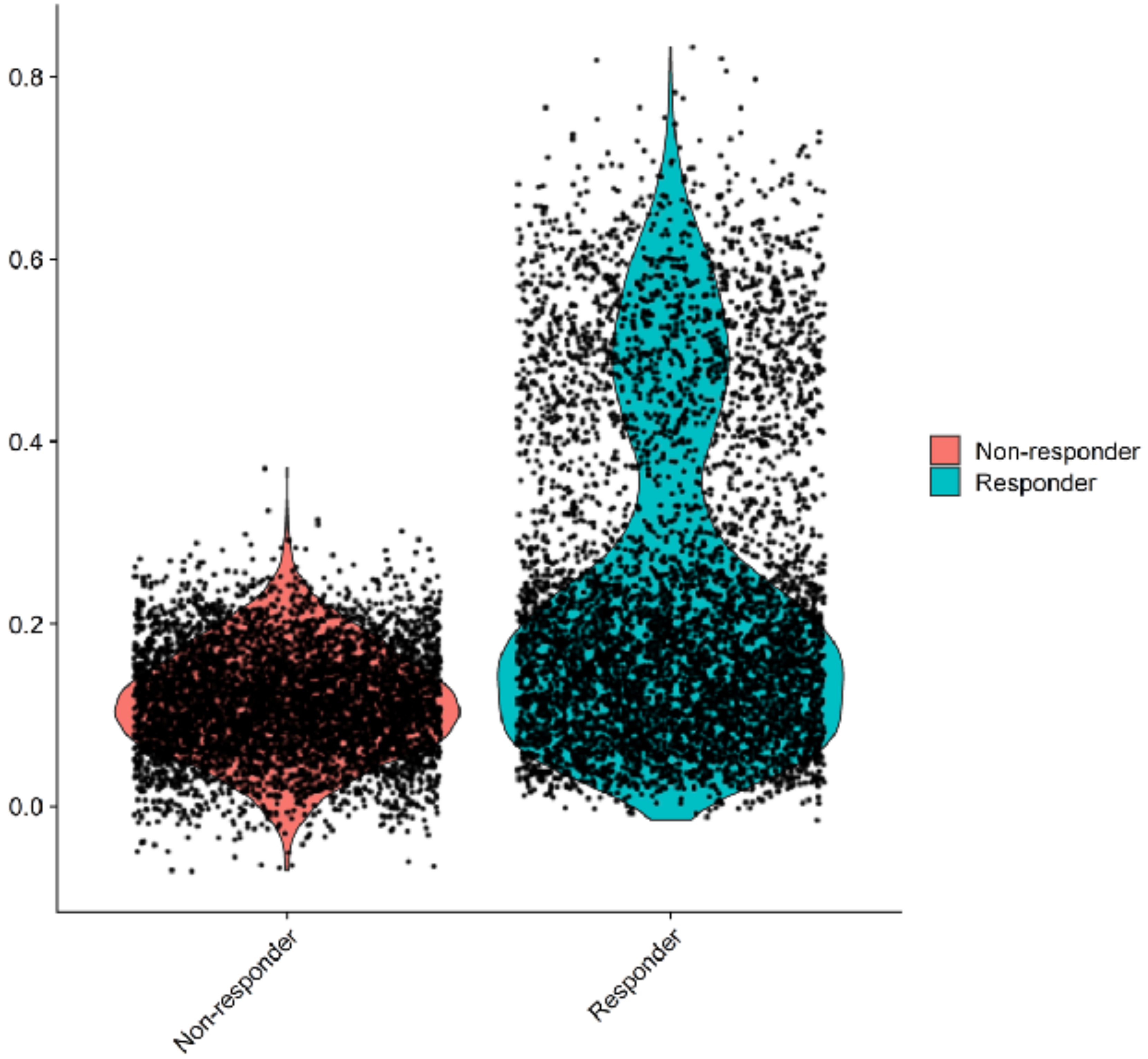

Fig. S2 - Overall expression of the antigen processing and presentation signaling pathway in the subset of spots in the spatial transcriptomics data from non-responders and responders. Using the ModuleScore function from the Seurat Bioconductor/R package, the expression of all the genes in the KEGG antigen processing and presentation pathway was calculated in each spot assigned to tumor regions and a score representative of the averaged expression was determined. The module scores are increased in a significant proportion of tumor spots from responders, suggesting that immune cell recruitment was more efficient in the patients with pathological responses to the neoadjuvant therapy.
