# Supplementary material for: Spatial transcriptomics analysis of neoadjuvant cabozantinib and nivolumab in advanced hepatocellular carcinoma identifies independent mechanisms of resistance and recurrence
Source: Genome Med. 2023 Sep 18;15:72. doi: 10.1186/s13073-023-01218-y (PMC10506285; doi:10.1186/s13073-023-01218-y)
Supplement: Supplementary file 4 — Additional file 4: Fig. S3. Representation of adjacent regions selection for interaction analysis in one HCC sample. [file 13073_2023_1218_MOESM4_ESM.pdf]

Fig. S3

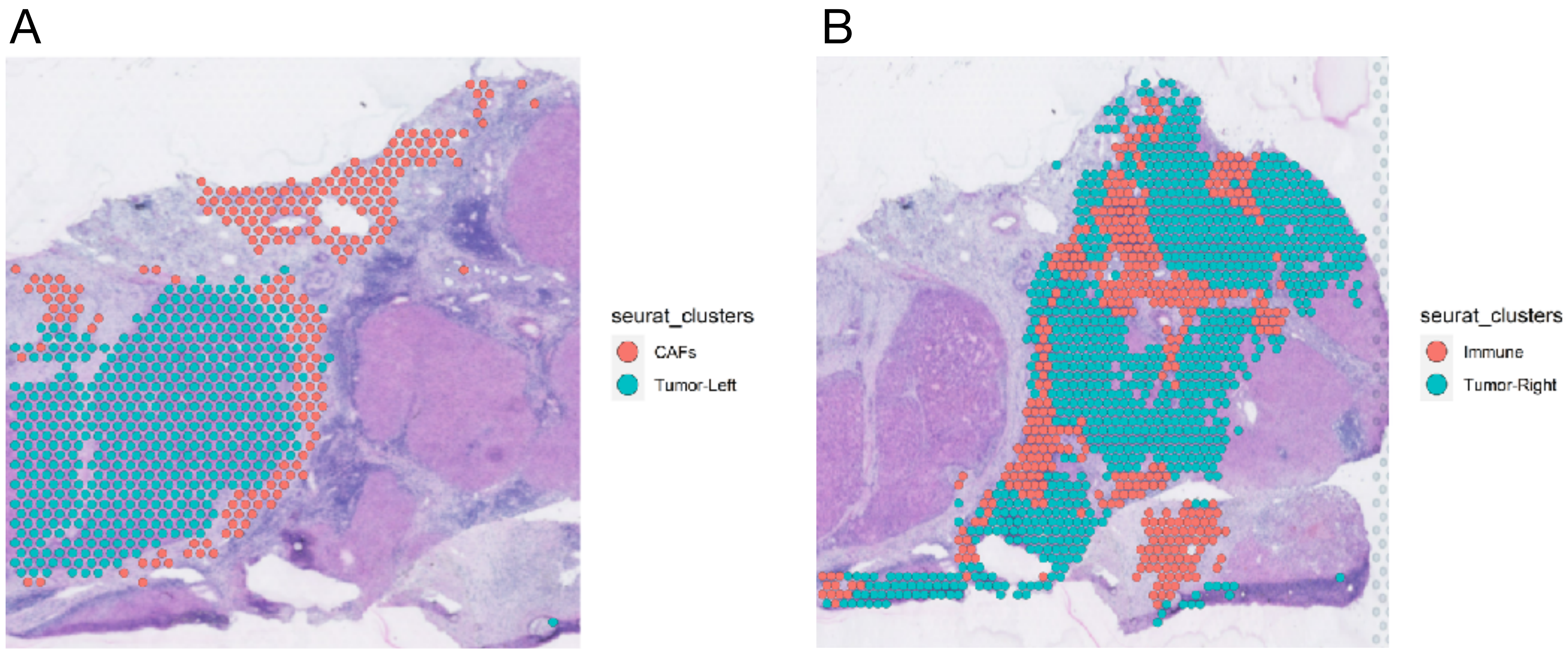

Fig. S3 - Representation of adjacent regions selection for interaction analysis in one HCC sample. CAF (A) and immune (B) regions, in red, adjacent (in direct contact) with tumor clusters (cyan) were selected for the interaction analysis with DOMINO. The strategy was repeated across all samples in the study.
