# Supplementary material for: Spatial transcriptomics analysis of neoadjuvant cabozantinib and nivolumab in advanced hepatocellular carcinoma identifies independent mechanisms of resistance and recurrence
Source: Genome Med. 2023 Sep 18;15:72. doi: 10.1186/s13073-023-01218-y (PMC10506285; doi:10.1186/s13073-023-01218-y)
Supplement: Supplementary file 5 — Additional file 5: Fig. S4. Heatmap of transcription factor network scores from patient HCC3-R as a representation of the DOMINO networks obtained from the interaction analysis. [file 13073_2023_1218_MOESM5_ESM.pdf]

Fig. S4

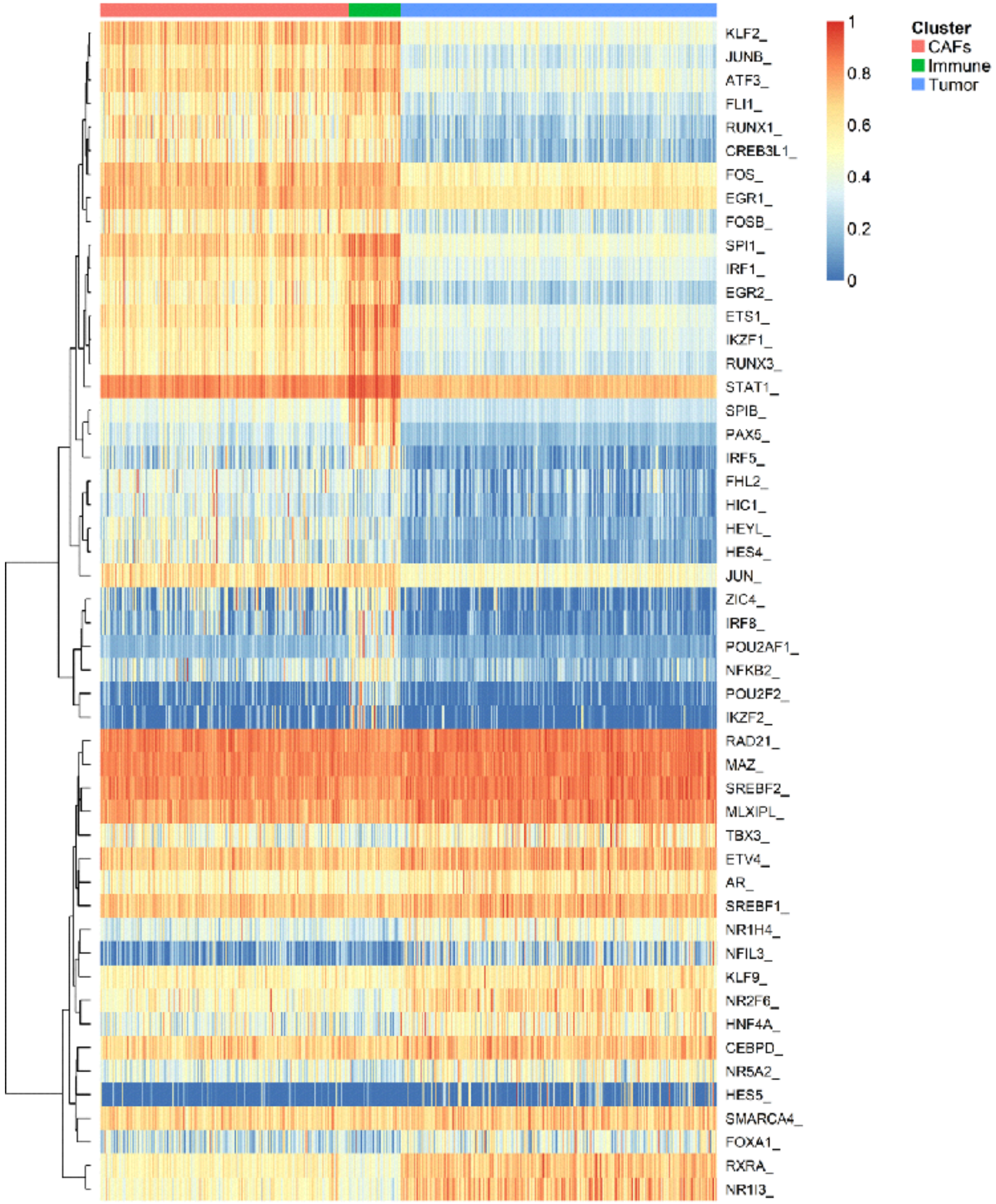

Fig. S4 - Heatmap of transcription factor network scores from patient HCC3-R as a representation of the DOMINO networks obtained from the interaction analysis. Each column represents a single spot from the spatial transcriptomics data and the rows represent the transcription factor networks. Spots are grouped by spatial clusters (cell types).
