# Supplementary material for: Spatial transcriptomics analysis of neoadjuvant cabozantinib and nivolumab in advanced hepatocellular carcinoma identifies independent mechanisms of resistance and recurrence
Source: Genome Med. 2023 Sep 18;15:72. doi: 10.1186/s13073-023-01218-y (PMC10506285; doi:10.1186/s13073-023-01218-y)
Supplement: Supplementary file 6 — Additional file 6: Fig. S5. Differential expression analysis between the immune poor and immune rich regions on sample from patient HCC1-R. [file 13073_2023_1218_MOESM6_ESM.pdf]

Fig. S5

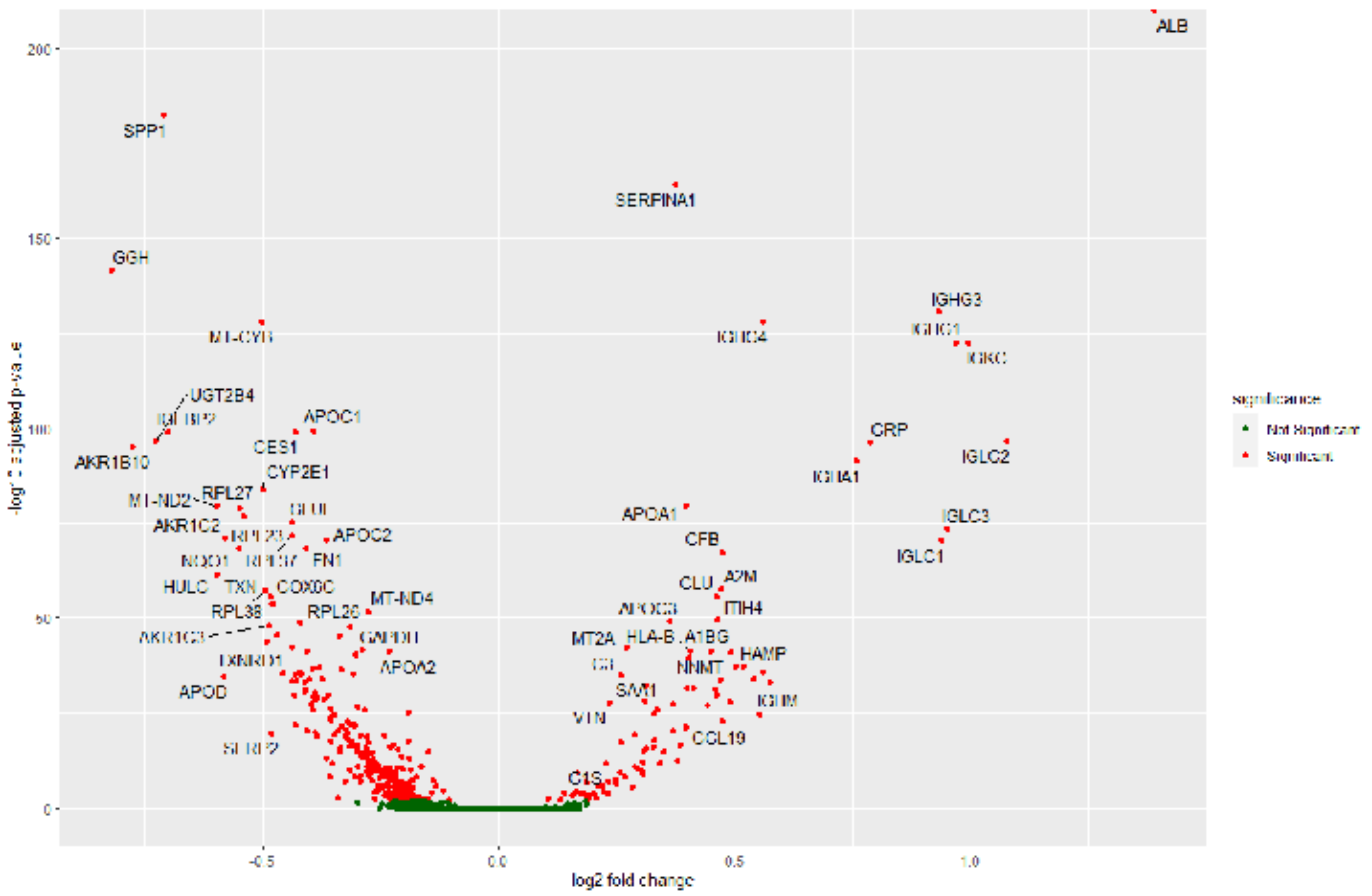

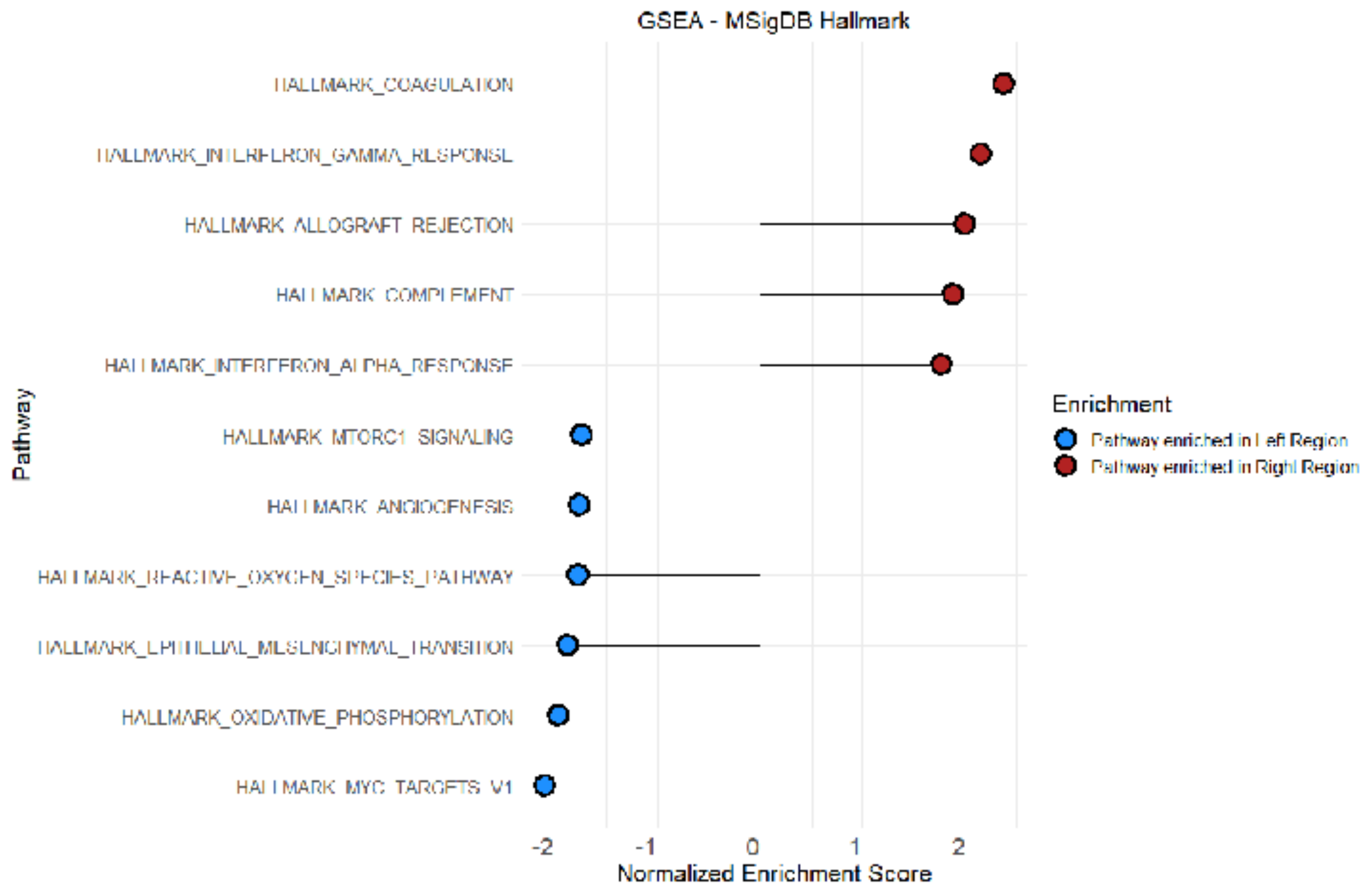

Fig. S5 - Differential expression analysis between the immune poor and immune rich regions on sample from patient HCC1-R. The volcano plot (left panel) represents the up-regulated genes in the immune poor tumor region (genes on the left) and in the immune rich tumor region (genes on the right). Most of the markers in the immune poor region are HCC or common tumor markers while the immune rich region up-regulates immune related genes. The gene set enrichment analysis (GSEA - right panel) revealed that the immune rich tumor expresses genes associated with immune response, while the immune poor tumor expresses genes from metabolic and cell proliferation pathways.
