# Supplementary material for: Spatial transcriptomics analysis of neoadjuvant cabozantinib and nivolumab in advanced hepatocellular carcinoma identifies independent mechanisms of resistance and recurrence
Source: Genome Med. 2023 Sep 18;15:72. doi: 10.1186/s13073-023-01218-y (PMC10506285; doi:10.1186/s13073-023-01218-y)
Supplement: Supplementary file 8 — Additional file 8: Fig. S6. Expression of MSigDB Hallmark pathways across all spots on the immune poor and immune rich tumor regions from patient HCC1-R. [file 13073_2023_1218_MOESM8_ESM.pdf]

Fig. S6

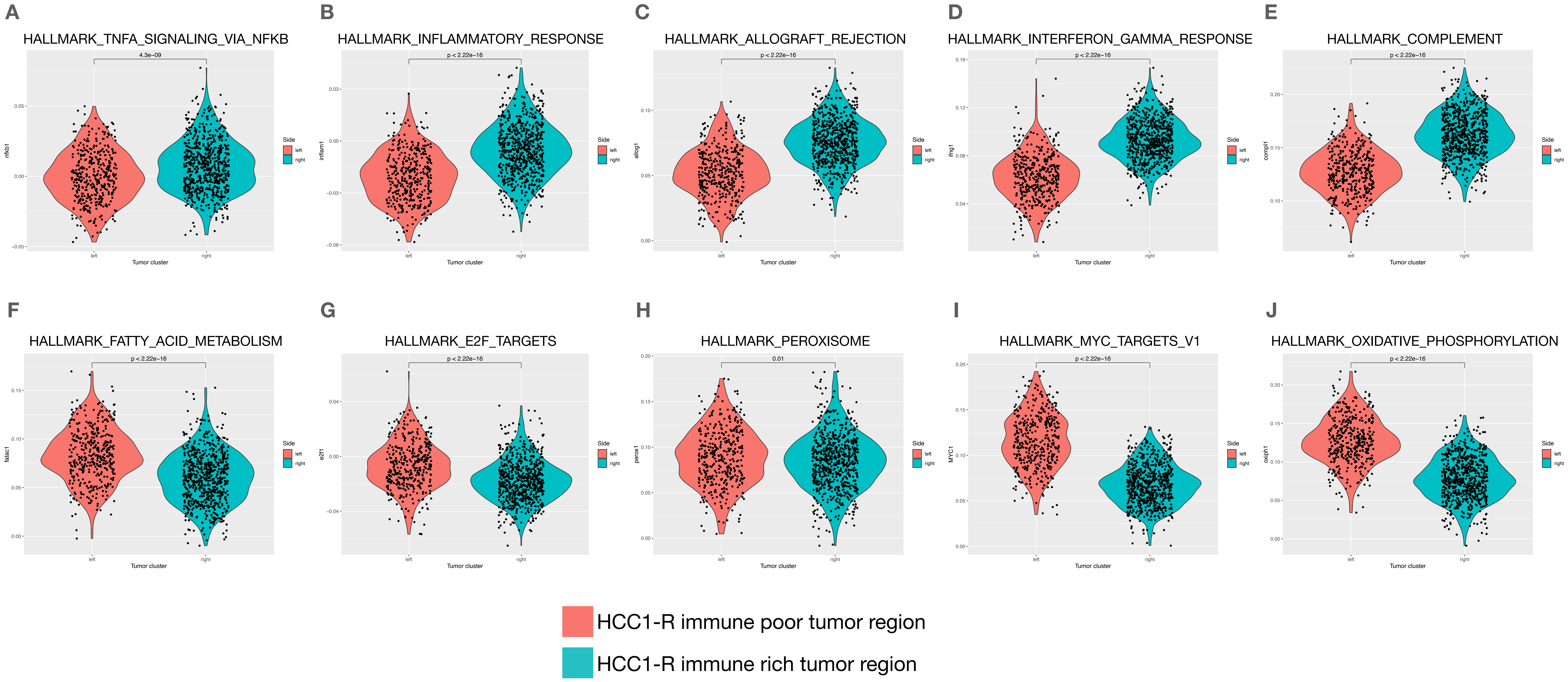

Fig. S6 - Expression of MSigDB Hallmark pathways across all spots on the immune poor and immune rich tumor regions from patient HCC1-R. A, B, C, D and E represent the module score expression distribution of immune related pathways in HCC1-R immune rich regions that are enriched in responder samples according to gene set enrichment analysis. F, G, H, I and J represent the module score expression distribution of proliferation and metabolic related pathways in HCC1-R immune poor regions that are enriched in non-responder samples according to gene set enrichment analysis.
