# Supplementary material for: Spatial transcriptomics analysis of neoadjuvant cabozantinib and nivolumab in advanced hepatocellular carcinoma identifies independent mechanisms of resistance and recurrence
Source: Genome Med. 2023 Sep 18;15:72. doi: 10.1186/s13073-023-01218-y (PMC10506285; doi:10.1186/s13073-023-01218-y)
Supplement: Supplementary file 9 — Additional file 9: Fig. S7. Heatmap of transcription factor network scores from patient HCC1-R from the DOMINO interaction analysis. [file 13073_2023_1218_MOESM9_ESM.pdf]

Fig. S7

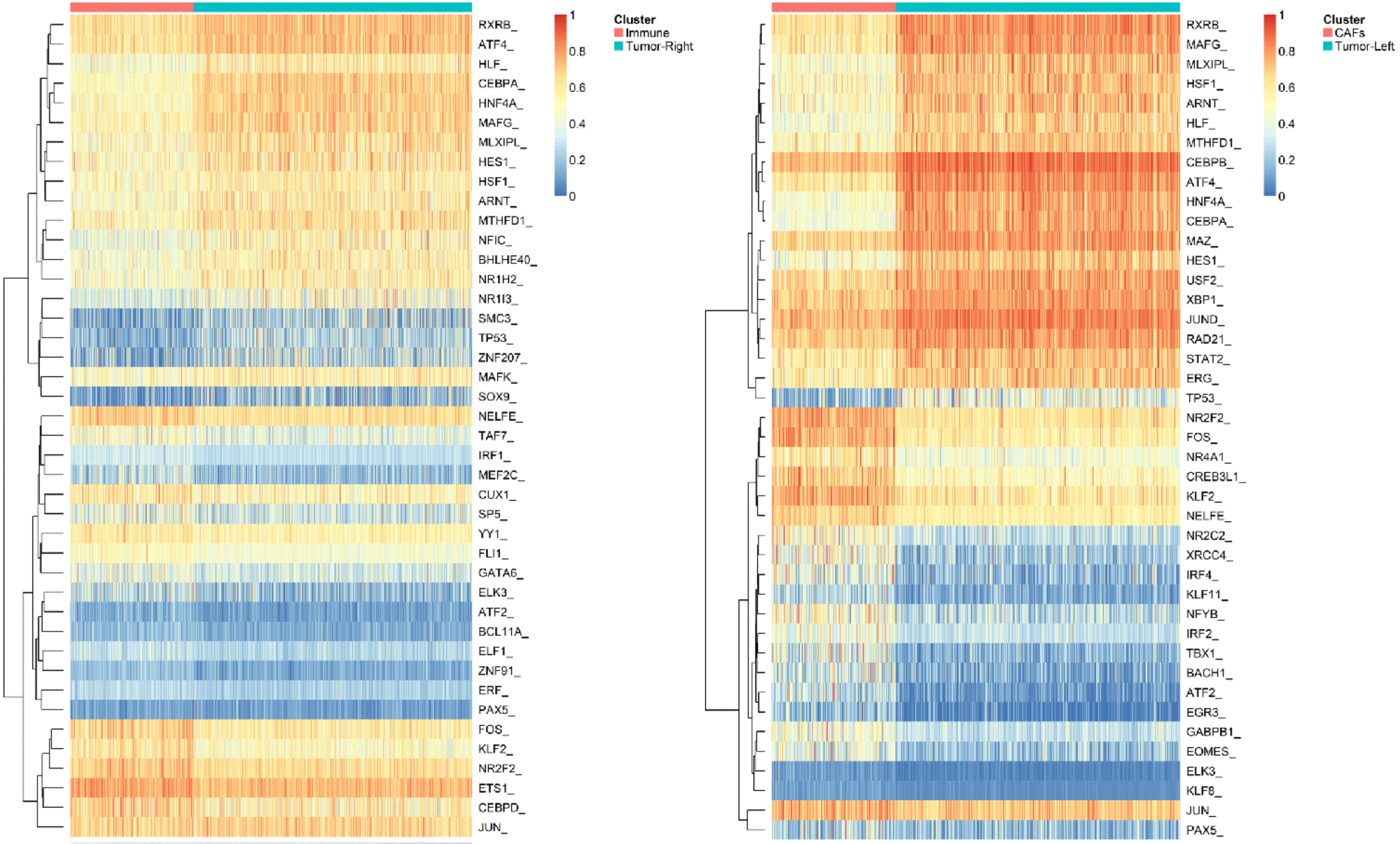

Fig. S7 - Heatmap of transcription factor network scores from patient HCC1-R from the DOMINO interaction analysis. On the left, the heatmap represents the networks that are active in the tumor and immune adjacent clusters. On the right is the heatmap with the activation scores for the networks obtained with the interaction analysis of CAF and tumor cells. Each column represents a single spot from the spatial transcriptomics data and the rows represent the transcription factor networks. Spots are grouped by spatial clusters (cell types).
