# Supplementary material for: Spatial transcriptomics analysis of neoadjuvant cabozantinib and nivolumab in advanced hepatocellular carcinoma identifies independent mechanisms of resistance and recurrence
Source: Genome Med. 2023 Sep 18;15:72. doi: 10.1186/s13073-023-01218-y (PMC10506285; doi:10.1186/s13073-023-01218-y)
Supplement: Supplementary file 10 — Additional file 10. [file 13073_2023_1218_MOESM10_ESM.pdf]

TITLE: Feasibility and Efficacy of Neoadjuvant Cabozantinib plus Nivolumab (CaboNivo) Followed by Definitive Resection for Patients with Locally Advanced Hepatocellular Carcinoma (HCC)

**Johns Hopkins Protocol #:**	J17136/IRB00149350
**BMS Protocol #:**	CA209-9L4
**Exelixis Protocol #:**	IST55
**Principal Investigator:**	Mark Yarchoan, M.D.<br>1650 Orleans St. CRB1 406<br>Baltimore, MD 21287<br>410-955-8893<br>mark.yarchoan@jhmi.edu
**IND#:**	IND 136943
**IND Sponsor:**	Daniel Laheru, M.D.
**Statistician:**	Elizabeth Sugar, MS, PhD, 410-614-7837, esugar@jhsph.edu
**Program Manager:**	Katrina Purtell, RN, BSN, Kalino1@jhmi.edu
**Lead Research Nurse:**	Brad Wilt, RN, BSN, 410-614-1816, bwilt1@jhmi.edu
**Lead Study Coordinator:**	Tearra Miles, 410-502-5328, tlawre18@jhmi.edu
**Regulatory:**	Jennifer Durham, PhD, 410-502-3038, juram1@jhmi.edu<br>Rose Parkinson, PhD, 443-287-8568, rparkin1@jhmi.edu
**Research Facility:**	The Johns Hopkins Oncology Center<br>600 N. Wolfe Street, Baltimore, MD 21287-7509
**IRB:**	Johns Hopkins Medicine- Institutional Review Board<br>1620 McElderry Street, Reed Hall, Suite B-130<br>Baltimore, MD 21205-1911

**Version:**	Original/ Version 1.0/August 23, 2017<br>Original/ Version 1.1/October 12, 2017<br>Original/ Version 1.2/October 27, 2017<br>Amendment 1/Version 2.0/January 22, 2018<br>Amendment 2/Version 3.0/July 15, 2018<br>Amendment 3/Version 4.0/November 15, 2018<br>Amendment 4/Version 5.0/May 13, 2019<br>Amendment 5/Version 6.0/March 11, 2020<br>Amendment 6/Version 7.0/February 2, 2021

1. SCHEMA

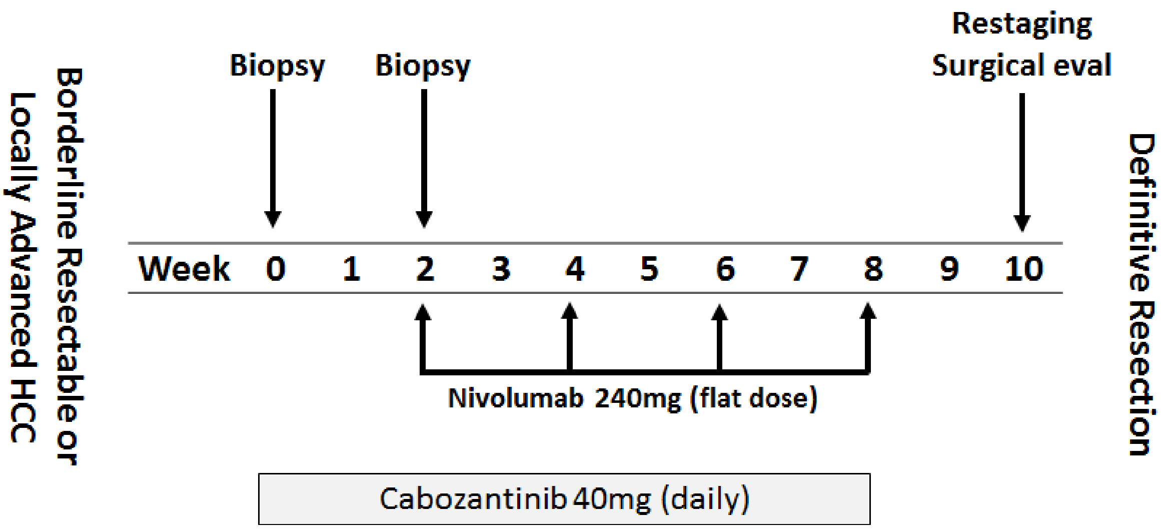

***Figure 1**: Overview of the treatment plan for subjects who are enrolled in this clinical trial.*

TABLE OF CONTENTS

1. SCHEMA 3

2. OBJECTIVES and endpoints 6
   2.1 Primary Objective 6
   2.2 Secondary Objectives 6
   2.3 Exploratory Objectives 6
   2.4 Primary Endpoints 6
   2.5 Secondary Endpoints 6
   2.6 Exploratory Endpoints 7
   2.7 Study Design 7

3. BACKGROUND 8
   3.1 Study Disease 8
   3.2 Interventions/therapy agents 8
   3.3 Rationale 14

4. PATIENT SELECTION 15
   4.1 Eligibility Criteria 15
   4.2 Exclusion Criteria 17
   4.3 Inclusion of Women and Minorities 19

5. TREATMENT PLAN 19
   5.1 Agent Administration 20
   5.2 General Concomitant Medication and Supportive Care Guidelines 21
   5.3 Prohibited and Restricted Therapies 22
   5.4 Definition of an Overdose for this Protocol 25
   5.5 WOCBP, Contraception, Use in Pregnancy, Use in Nursing: 25
   5.6 Unacceptable Toxicities 28
   5.7 Criteria for Removal from Treatment 28
   5.8 End of Treatment (EOT) Visit 29
   5.9 Duration of Follow Up 29

6. DOSING DELAYS/DOSE MODIFICATIONS 30
   6.1 Dosing Criteria and General Dose Modifications for Cabozantinib 30
   6.2 Dosing Criteria for Nivolumab 31

7. ADVERSE EVENTS: LIST AND REPORTING REQUIREMENTS 32
   7.1 Definitions 33
   7.2 Relationship and Grading 34
   7.3 Expectedness 35
   7.4 Handling of Expedited Safety Reports 35
   7.5 Reporting 36
   7.6 Special considerations for adverse events that occur during the surgery and postoperative course 38

8. BIOMARKER, CORRELATIVE, AND SPECIAL STUDIES 39
   8.1 Whole blood 39

8.2 Tumor Tissue Studies 39

9. STUDY CALENDAR 41

10. MEASUREMENT OF EFFECT..42
10.1 Definitions..42

11. DATA REPORTING / REGULATORY REQUIREMENTS..44
11.1 Data Quality Assurance and Management..44
11.2 Safety Meetings 44
11.3 Monitoring 44

12. Presentation of Study Data and Study Termination..44
12.1 Publication of Data and Protection of Trade Secrets..44
12.2 Conditions for Terminating the Study 45

13. STATISTICAL CONSIDERATIONS..45
13.1 Primary Endpoint..45
13.2 Safety Endpoints..45
13.3 Sample Size/Accrual Rate..47
13.4 Stratification Factors..48
13.5 Analysis of Secondary and Exploratory Endpoints 48
13.6 Reporting and Exclusions 48

REFERENCES 50

APPENDIX A PERFORMANCE STATUS CRITERIA 52

APPENDIX B PATIENT DRUG INFORMATION HANDOUT AND WALLET CARD 53

APPENDIX C PATIENT MEDICATION DIARY..56

APPENDIX D RECOMMENDED MANAGEMENT ALGORITHMS..60

APPENDIX E SAE REPORTING FORM..72

APPENDIX F RESPONSE EVALUATION CRITERIA IN SOLID TUMORS (RECIST) 1.1 CRITERIA FOR EVALUATING RESPONSE IN SOLID TUMORS..74

APPENDIX G IMMUNE RELATED RESPONSE CRITERIA (irrc) CRITERIA FOR EVALUATING RESPONSE IN SOLID TUMORS..77

2. OBJECTIVES AND ENDPOINTS

2.1 Primary Objective

2.1.1 To characterize the safety and feasibility of preoperative cabozantinib plus nivolumab in locally advanced hepatocellular carcinoma (HCC).

2.2 Secondary Objectives

2.2.1 To estimate the percentage of patients with locally advanced HCC who obtain an R0 resection at week 12 following neoadjuvant cabozantinib plus nivolumab.

2.2.2 To determine the percentage of evaluable patients who obtain a pathologic complete response (CR) or major pathologic responses (MPR) at week 12 following neoadjuvant cabozantinib plus nivolumab.

2.2.3 To determine the objective response rate (ORR) at week 8 in patients with locally advanced HCC treated with neoadjuvant cabozantinib plus nivolumab.

2.2.4 To determine the median overall survival (OS), 12 month OS, 18 month OS, 3 year OS, and 5 year OS, of patients with locally advanced HCC treated with neoadjuvant cabozantinib plus nivolumab.

2.2.5 To determine the median disease free survival (DFS), 12 month DFS, 18 month DFS, 3 year DFS, and 5 year DFS, of subjects with locally advanced HCC who obtain a successful definitive resection following neoadjuvant cabozantinib plus nivolumab.

2.3 Exploratory Objectives

2.3.1 To explore the effects of therapy on tumor and peripheral blood and tumor infiltrating immune cells, and to explore potential molecular determinants of response, progression and disease stability.

2.4 Primary Endpoints

2.4.1 Feasibility will be assessed based upon the number of treatment failures, defined as individuals with an extended treatment-related delay, defined as >60 days from preplanned surgical evaluation date in this context. Individuals who do not successfully complete preoperative treatment or do not proceed to surgery (e.g. patient refusal, not a surgical candidate) will not be considered treatment failures.

Safety will be assessed by the frequency of adverse events occurring up to 100 days after the last dose of nivolumab.

2.5 Secondary Endpoints

2.5.1 Percentage of subjects who receive an R0 surgical resection at week 12 following neoadjuvant cabozantinib plus nivolumab.

2.5.2 pCR rate, as defined by no residual cancer in the surgical resection specimen obtained at week 12; and MPR rate, as defined by <10% residual viable tumor in the surgical resection obtained at week 12

2.5.3 ORR at week 8, defined as the proportion of patients achieving a complete response (CR) or partial response (PR) based on the Response Evaluation Criteria in Solid Tumors (RECIST 1.1) and immune-related response criteria (irRC).

2.5.4 The overall survival (OS)

2.5.5 Disease free survival (DFS), defined as the time from surgery to recurrence, or death.

2.6 Exploratory Endpoints

2.6.1 Peripheral blood specimens, intratumoral core biopsy specimens, and resection specimens will be studied using a variety of laboratory techniques including but not limited to: immunohistochemistry (IHC), flow cytometry, CITE-Seq, RNA-Seq, whole exome sequencing, whole genome sequencing, T cell receptor and B cell receptor sequencing, ChIP-seq, ATAC-seq, and MBD-seq.

2.7 Study Design

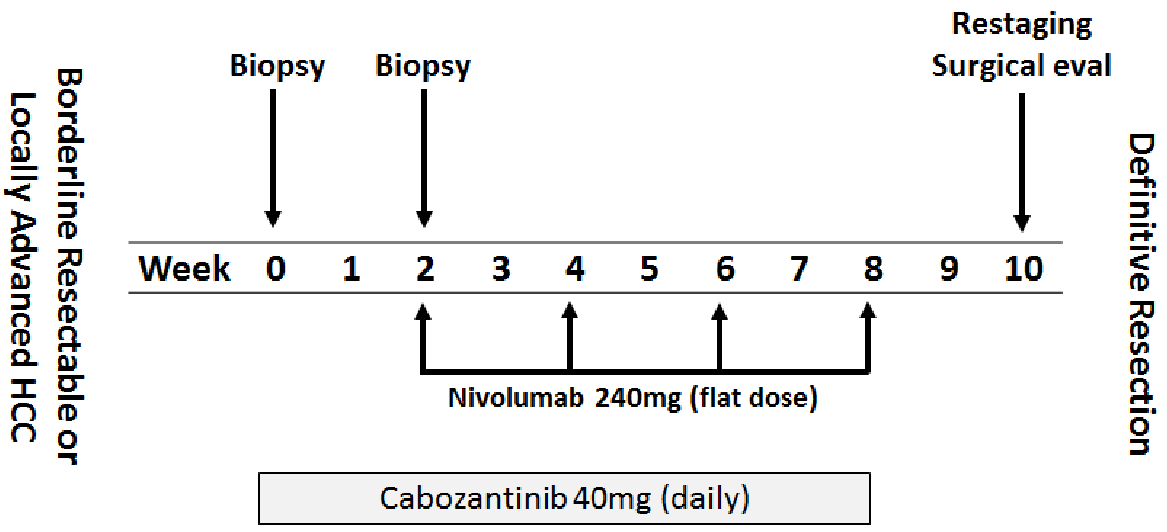

This is an open-label, single institution, single arm phase 1b study of neoadjuvant cabozantinib plus nivolumab in patients with locally advanced HCC. The purpose of this study is to determine the safety and tolerability of neoadjuvant cabozantinib plus nivolumab in patients with HCC. Patients with HCC that is potentially resectable but at high risk of disease recurrence are eligible for this clinical trial. Enrolled patients will receive a total of 8 weeks of cabozantinib therapy. Patients will receive a biopsy prior to starting therapy and at the end of the 2-week cabozantinib monotherapy lead in. After a two-week lead-in of cabozantinib monotherapy, patients will receive concurrent nivolumab, one infusion every 2 weeks, for a total of 4 treatment doses.

Two weeks after completing neoadjuvant therapy consisting of 8 weeks of cabozantinib and 4 doses of nivolumab, patients will receive a restaging scan and surgical evaluation. Patients who are eligible for surgical resection will proceed to a definitive surgical resection. In order to mitigate risk of severe hemorrhage resulting from cabozantinib, the surgical resection should occur at least 28 days after the last dose of cabozantinib therapy. The surgical resection should be scheduled no more than 60 days after surgical evaluation.

3. BACKGROUND

3.1 Study Disease

Hepatocellular carcinoma (HCC) is a cancer of the liver that usually develops in the setting of chronic liver disease including alcohol, chronic viral hepatitis (B or C), and non-alcoholic fatty liver disease. HCC is the second leading cause of cancer-related death in the world, and the incidence of HCC is rising in many parts of the world (Park *et al*, 2015). For all patients with HCC, the historical median overall survival is less than 2 years.

Patients with small (<5 cm), potentially resectable tumors have the potential for favorable long term survival with a partial hepatectomy or liver transplantation. However, the outcomes for patients with larger tumors that are either technically resectable or not resectable is much less favorable. Some of these patients receive palliative treatments [for example, transarterial chemoembolization (TACE)]. Others receive a partial resection, but unfortunately these patients have a very high rate of intrahepatic disease relapse. For patients receiving a partial hepatectomy for HCC that is greater than 5 cm, the 5-year survival rate was 37.1% in one series (Zhou *et al*, 2001). The optimal management of these patients remains unclear.

We hypothesize that the use of neoadjuvant therapy patients with locally advanced HCC will result in tumor response and downsizing, increased probability of successful surgical resection, and improved long-term outcomes. In this study, we are investigating whether neoadjuvant therapy for locally advanced HCC is safe and feasible.

3.2 Interventions/therapy agents

3.2.1 <u>Overview of Cabozantinib (CABOMETYX®, COMETRIQ®, XL184)</u>

Chemical Name: Chemical Name: N-{4-[(6,7-dimethoxyquinolin-4-yl)oxy]phenyl}-N'-(4-fluorophenyl)cyclopropane-1,1-dicarboxamide, (2S)-hydroxybutanedioate

Cabozantinib is an orally bioavailable tyrosine kinase inhibitor with potent activity against MET and VEGFR2, as well as a number of other receptor tyrosine kinases that have also been implicated in tumor pathobiology, including RET, KIT, AXL, and FLT3. Cabozantinib suppresses MET and VEGFR2 signaling, rapidly inducing apoptosis of endothelial and tumor cells, resulting in tumor regression in a variety of xenograft models. Cabozantinib prolonged survival in a MET-driven transgenic mouse model of HCC. In clinical studies, cabozantinib has demonstrated promising activity in multiple tumor types

and has been approved by the US FDA for the treatment of metastatic medullary thyroid cancer and metastatic renal cell carcinoma. It is under phase 3 investigation for the treatment of advanced HCC previously treated with sorafenib.

3.2.2 Cabozantinib Tablet Components and Composition

All study medication will be stored as described in the appropriate prescribing information for that country (if applicable) or the pharmacy manual and inventoried in accordance with applicable state and federal regulations

Cabozantinib tablets are supplied as film coated tablets containing cabozantinib malate equivalent to 20 mg of cabozantinib and contain microcrystalline cellulose, lactose anhydrous, hydoxypropyl cellulose, croscarmellose sodium, colloidal silicon dioxide, magnesium stearate and Opadry® yellow. All tablet strengths are prepared from a common blend and are distinguished by shape. The 20 mg tablets are round. The components of the tablets are listed in **Table 1**.

Table 1: Cabozantinib composition

Ingredient	Function	% by weight
Cabozantinib malate (25% drug load as cabozantinib)	Active Ingredient	31.7
Microcrystalline Cellulose (Avicel PH-102)	Filler	38.9
Lactose Anhydrous (60M)	Filler	19.4
Hydroxypropyl Cellulose (EXF)	Binder	3.0
Croscarmellose Sodium (Ac-Di-Sol)	Disenegrant	6.0
Colloidal Silicon Dioxide,	Glidant	0.3
Magnesium Stearate	Lubricant	0.75
Opadry Yellow Film Coating which includes:<br>- HPMC 2910/Hypromellose 6 cp<br>- Titanium dioxide<br>- Triacetin<br>- Iron Oxide Yellow	Film Coating	4.00

3.2.3 Pharmacology of Cabozantinib

Cabozantinib exhibits potent inhibitory activity against several receptor tyrosine kinases that are known to influence tumor growth, metastasis, and angiogenesis. The primary targets of cabozantinib are MET, VEGFR2/KDR, and RET with cell-based IC50 (concentration associated with 50% inhibition) values of 8, 2 and 85 nM, respectively. In addition, cabozantinib inhibited phosphorylation of KIT, FLT3, and AXL with IC50 values of 5, 11, and 42 nM, respectively.

3.2.4 Safety and Efficacy of Cabozantinib in Hepatocellular Carcinoma (Study XL184-203)

The 41 subjects with advanced HCC treated with cabozantinib in Study XL184-203 received an initial dose of 100 mg/day (FBE). Fifty-nine percent of subjects required at least 1 dose reduction throughout both the Lead-in and Randomized Stages.

The most frequently reported AEs during the study were consistent with those in subjects with other tumor types who received single-agent cabozantinib and included diarrhea (68%), fatigue (59%), palmar-plantar erythrodysesthesia (PPE) syndrome (54%), vomiting (42%), and nausea (39%). Common Grade 3 or higher AEs included diarrhea (22%), thrombocytopenia (17%), PPE syndrome (15%), aspartate aminotransferase (AST) increased (12%).

Two subjects (5%) had a confirmed partial response (PR) during the 12-week Lead-in Stage (at any time through Week 12) and 31 subjects (76%) had stable disease; the disease control rate (PR plus stable disease) at Week 12 was 66%. One subject with stable disease at Week 12 subsequently achieved a partial response. The 3 subjects with PRs were White; one each with HCC etiology of Hepatitis C, Hepatitis B, and Alcoholism, respectively. Twenty-eight of 36 subjects (78%) had at least 1 scan demonstrating a reduction in measurable disease, sufficient in 2 subjects to be considered a partial response (four subjects were not evaluable and the target lesion for the fifth subject became non-measurable post-baseline). Regression appeared independent of prior sorafenib exposure.

In a preliminary analysis of the data, twenty-six subjects were evaluable for alpha-fetoprotein (AFP) changes, namely, with a baseline value greater than 20 ng/mL and at least 1 post-baseline measurement. Of these, 9 subjects (35%) demonstrated a decrease by at least 50% during the initial 12 weeks of therapy and an additional 7 subjects (27%) showed some degree of reduction.

Twenty-two of the 41 subjects enrolled in the lead-in stage were randomized at Week 12 to either placebo or continuing cabozantinib after demonstrating stable disease. Median PFS for all subjects from the initial cabozantinib dose was 5.2 months by Kaplan-Meier estimate and did not appear to be influenced by sorafenib pretreatment status (5.2 months for sorafenib pre-treated subjects [n=22] and 4.2 months for sorafenib naïve subjects [n=19]). No statistically significant difference in median PFS between randomized treatment groups was observed from the point of randomization: median PFS was 1.4 months (95% CI: 1.3, 4.2) for placebo and 2.5 months (95% CI: 1.3, 6.8) for cabozantinib. The median OS for all treated patients (n=41) from the initial cabozantinib dose as estimated by the Kaplan-Meier method was 11.5 months (95% CI: 7.3, 15.6).

3.2.5 Clinical Pharmacokinetics (PK) of Cabozantinib

A population PK analysis of cabozantinib was performed using data collected from 289 subjects with solid tumors including MTC following oral administration of 140 mg (FBE) daily doses. The predicted effective half-life is approximately 55 hours, the oral volume of distribution (V/F) is approximately 349 L, and the clearance (CL/F) at steady-state is estimated to be 4.4 L/hr. The terminal half-life (for predicting drug washout) is approximately 120 hours. Following oral administration of cabozantinib, median time to peak cabozantinib plasma concentrations (Tmax) ranged from 2 to 5 hours post-dose. Repeat daily dosing of cabozantinib at 140 mg for 19 days resulted in 4- to 5-fold mean cabozantinib accumulation (based on AUC) compared to a single dose administration; steady state was achieved by Day 15. Cabozantinib is highly protein bound in human plasma (≥ 99.7%).

A population PK analysis did not identify clinically relevant differences in clearance of cabozantinib between females and males or between Whites (89%) and non-Whites (11% [<4% were Asian]). Cabozantinib PK was not affected by age (20-86 years). Exposure of cabozantinib was assessed in Japanese subjects in capsule and tablet formulations (Study XL184-014). At steady state, exposure, AUC increased approximately dose proportionally from 40 to 80 mg capsule doses and from 40 to 60 mg tablet doses. Exposure between capsule and tablet formulations appeared to be similar. The exposure of cabozantinib at 40 and 60 mg doses in Japanese subjects from this study appeared to be slightly higher than the values observed in non-Japanese subjects.

In the mass balance study, within a 48-day collection period after a single dose of cabozantinib in healthy subjects, approximately 81% of the total administered radioactivity was recovered with 54% in feces and 27% in urine. A PK study of cabozantinib in patients with renal impairment is ongoing. The results of a population PK analysis suggested that mild to moderate renal impairment (creatinine clearance value ≥30 mL/min) does not have a clinically relevant effect on the clearance of cabozantinib.

A high-fat meal increased Cmax and AUC values by 41% and 57%, respectively relative to fasted conditions in healthy subjects administered a single 140 mg oral cabozantinib dose. Cabozantinib is a substrate of CYP3A4 in vitro. Inhibition of CYP3A4 reduced the formation of the XL184 N-oxide metabolite by >80%. Inhibition of CYP2C9 had a minimal effect on cabozantinib metabolite formation (i.e., a <20% reduction). Inhibition of CYP1A2, CYP2A6, CYP2B6, CYP2C8, CYP2C19, CYP2D6 and CYP2E1 had no effect on cabozantinib metabolite formation. Cabozantinib AUC was increased 38% with co-administration of the strong CYP3A4 inhibitor ketoconazole and decreased 77% with co-administration of the strong CYP3A4 inducer rifampin.

Cabozantinib is a noncompetitive inhibitor of CYP2C8 (Kiapp = 4.6 μM), a mixed-type inhibitor of both CYP2C9 (Kiapp = 10.4 μM) and CYP2C19 (Kiapp = 28.8 μM), and a weak competitive inhibitor of CYP3A4 (estimated Kiapp = 282 μM) in human liver microsomal (HLM) preparations. IC50 values >20 μM were observed for CYP1A2, CYP2D6, and CYP3A4 isozymes in both recombinant and HLM assay systems.

Cabozantinib is an inducer of CYP1A1 mRNA in human hepatocyte incubations (i.e., 75-100% of CYP1A1 positive control β-naphthoflavone induction), but not of CYP1A2, CYP2B6, CYP2C8, CYP2C9, CYP2C19 or CYP3A4 mRNA or isozyme-associated enzyme activities. Cabozantinib at steady-state plasma concentrations (≥100 mg/day daily for a minimum of 21 days) showed no effect on single-dose rosiglitazone (a CYP2C8 substrate) plasma exposure (Cmax and AUC) in patients with solid tumors. Cabozantinib is an inhibitor (IC50 = 7.0 μM), but not a substrate, of P-gp transport activities in a bi-directional assay system using MDCK-MDR1 cells. Therefore, cabozantinib may have the potential to increase plasma concentrations of co-administered substrates of P-gp.

3.2.6 Overview of Nivolumab (OPDIVO®, BMS-936558, MDX-1106)

Nivolumab is a potent and highly-selective monoclonal antibody that binds to the PD-1 receptor and blocks its interaction with PD-L1 and PD-L2, thereby releasing PD-1 pathway-mediated inhibition of the immune response, including anti-tumor immune

response. It is FDA-approved for treating metastatic melanoma, non-small cell lung cancer (NSCLC), metastatic kidney cancer, recurrent or metastatic squamous cell carcinoma of the head and neck, locally advanced or metastatic urothelial carcinoma, and Hodgkin Lymphoma. In this study, nivolumab will administered as an intravenous infusion (IV) over 60 minutes at 240mg (flat dose) using a volumetric pump with a 0.2/0.22 micron in-line filter at the protocol-specified dose. It is not to be administered as an IV push or bolus injection. At the end of the infusion, flush the line with a sufficient quantity of normal saline (approximately 30-50mL).

**Table 2: Nivolumab Product Information**				
Product Description and Dosage Form	Potency	Primary Packaging (Qty) /Label Type	Appearance	Storage Conditions (per label)
Nivolumab (BMS-936558-01)* Injection drug product is a sterile, non-pyrogenic, single-use, isotonic aqueous solution formulated at 10 mg/mL	100 mg/Vial (10 mg/mL).	10-cc Type 1 flint glass vials stoppered with butyl stoppers and sealed with aluminum seals.	Clear to opalescent, colorless to pale yellow liquid. May contain particles	BMS-936558-01 Injection must be stored at 2 to 8 degrees C (36 to 46 degrees F) and protected from light and freezing

BMS-936558-01 injection should be diluted with 0.9% Sodium Chloride Injection, USP or 5% Dextrose Injection, USP to protein concentrations as low as 0.35 mg/mL. Total infusion volume should not exceed 120mL. Care must be taken to assure sterility of the prepared solution as the product does not contain any anti-microbial preservative or bacteriostatic agent. No incompatibilities between nivolumab and polyolefin bags have been observed. Recommended safety measures for preparation and handling of nivolumab include laboratory coats and gloves. Detailed instructions on the preparation of nivolumab for administration will be provided in the Pharmacy Manual.

The administration of nivolumab infusion must be completed within 24 hours of preparation. If not used immediately, the infusion solution may be stored under refrigeration conditions (2°-8°C, 36°-46°F) for up to 24 hours, and a maximum of 8 hours of the total 24 hours can be at room temperature (20°-25°C, 68°-77°F) and room light. The maximum 8-hour period under room temperature and room light conditions includes the product administration period.

3.2.7 Pharmacology of Nivolumab

Nivolumab (BMS-936558) is a fully human, IgG4 (kappa) isotype monoclonal antibody that binds to PD-1 with nanomolar affinity (KD = 3.06 nM) and a high degree of specificity, thus precluding binding to its ligands PD-L1 and PD-L2. Nivolumab (BMS-936558) does not bind other related family members, such as BTLA, CTLA-4, ICOS or CD28. Pre-clinical testing of nivolumab (BMS-936558) demonstrated that binding to PD-1 results in enhanced T cell proliferation and release of interferon-gamma (IFN-gamma) in vitro.

3.2.8 Clinical Pharmacokinetics (PK) of Nivolumab

A single dose pharmacokinetic analysis of 39 subjects with cancer given nivolumab (BMS936558) at 0.3, 1, 3 and 10 mg/kg revealed that the median Tmax across single doses ranged from 1.6 to 3 hours with individual values ranging from 0.9 to 7 hours. The pharmacokinetics of nivolumab (BMS-936558) were linear in the range of 0.3 to 10 mg/kg with dose proportional increases in maximum serum concentration (Cmax) and area under the concentration-time curve from time zero to infinity (AUCINF), with low to moderate inter-subject variability observed at each dose level. The mean terminal elimination half-life of nivolumab (BMS-936558) was 17 to 25 days, which is consistent with the half-life of endogenous IgG4. Both the elimination and distribution of nivolumab (BMS-936558) were independent of the dose.

3.2.9 Safety of Nivolumab

The overall safety experience with nivolumab, as monotherapy or in combination with other therapeutics, is based on experience in multiple large clinical trials to date. Overall, the safety profile of nivolumab monotherapy as well as combination therapy is manageable and generally consistent across completed and ongoing clinical trials with no MTD reached at any dose tested, up to 10 mg/kg. There was no pattern in the incidence, severity, or causality of AEs to nivolumab dose level. Most AEs were low grade (grade 1 to grade 2) with relatively few related high grade (grade 3 to grade 4) AEs. Most high-grade events were manageable with the use of corticosteroids or hormone replacement therapy for endocrinopathies.

For monotherapy, the safety profile is similar across tumor types. The one exception is pulmonary inflammation AEs which may be numerically greater in subjects with NSCLC because in some cases it can be difficult to distinguish between nivolumab related and unrelated causes of pulmonary symptoms and radiographic changes. There was no pattern in the incidence, severity, or causality of AEs to nivolumab dose level.

In several ongoing clinical trials, the safety of nivolumab in combination with other therapeutics such as ipilimumab, cytotoxic chemotherapy, anti-angiogenics and targeted therapies is being explored. Most studies are ongoing and as such, the safety profile of nivolumab combinations continues to evolve. The most advanced combination under development is nivolumab and ipilimumab in subjects with MEL. Thus far, the combination of both agents results in a safety profile with similar types of AEs as either agent alone, but in some cases with greater frequency.

3.2.10 Safety of Cabozantinib plus Nivolumab (CaboNivo)

The combination of cabozantinib plus nivolumab (CaboNivo) was assessed in a phase 1 clinical trial in patients with genitourinary tumors, and presented at the 2016 Annual Congress of the European Society for Medical Oncology in Copenhagen, Denmark (Apolo *et al*, 2016). A total of 24 patients were treated in this dose-escalation study (metastatic urothelial carcinoma N = 6; bladder urachal N = 4; bladder squamous cell carcinoma N = 3; germ cell tumor N = 5; castrate-resistant prostate cancer N = 4; renal cell carcinoma N = 1, and trophoblastic tumor N = 1). Four dose levels were studied (Cabo 40mg/Nivo 1mg/kg, DL2 Cabo 40mg/Nivo 3mg/kg, DL3 Cabo 60mg/Nivo1mg/kg, DL4 Cabo 60mg/Nivo 3mg/kg).

Overall CaboNivo was well tolerated, with no grade 4/5 toxicities and no DLTs. However, nine patients (38%) required dose reductions. The most common treatment related adverse events were transaminitis N = 20, diarrhea N = 11, hoarseness N = 7, dysgeusia N = 5, thrombocytopenia N = 5, hyponatremia N = 5. Grade 3 AEs included neutropenia N = 3 (DL1), fatigue N = 2 DL2, mucositis N = 1 (DL4), vomiting N = 1 (DL3).

The optimal dose for further study was determined to be cabozantinib 40mg and nivolumab 3mg mg/kg. An expansion study is ongoing, as well as a phase 1 triplet study with ipilimumab (CaboIpiNivo).

3.3 Rationale

Hepatocellular carcinoma (HCC) is an aggressive cancer and is the second leading cause of cancer-related death in the world (Park *et al*, 2015). Partial hepatectomy or liver transplantation are the only potentially curative treatments for HCC, but many patients are not eligible for curative therapy because of metastasis, local intrahepatic invasion, or comorbidities and/or poor hepatic reserve.

Even among the subset of patients who undergo a potentially curative partial hepatectomy, long-term outcomes are poor. Although estimates vary, recurrence rates of 54-100% have been reported in historical series of patients receiving a partial hepatectomy with curative intent for HCC (Belghiti *et al*, 1991; Chen *et al*, 1994; Lise *et al*, 1998; Portolani *et al*, 2006; Tabrizian *et al*, 2015). The majority of recurrences (~80%) are intrahepatic. Since negative margins are usually observed at the time of surgical resection, it is believed that HCC recurrence occurs as a result of micrometastasis that persist after resection. Therefore, neoadjuvant therapies that can reduce the burden of micrometastatic disease prior to resection have the potential to improve outcomes in this disease.

Neoadjuvant therapy may also improve outcomes by shrinking tumors and thereby converting patients with unresectable disease due to local invasion into surgical candidates. Our group at Johns Hopkins recently treated a patient with unresectable HCC due to local invasion with nivolumab (off label) after the patient had initially failed sorafenib therapy. The patient experienced a partial clinical response with a decrease in the size of his primary hepatic lesion, therefore qualifying him for a potentially curative partial hepatectomy. He had negative surgical margins upon surgical resection, demonstrating that some patients with unresectable HCC may become candidates for a potentially curative resection after successful neoadjuvant therapy. Additional support for neoadjuvant treatment in HCC comes from historical case series that have consistently demonstrated better surgical

outcomes and recurrence rates in patients with smaller tumors. In one historical case series, the 5-year survival rate was 62.7% for patients with a <5 cm lesion as compared to 37.1% for patients with a lesion >5 cm (Zhou *et al*, 2001). Therefore, neoadjuvant therapy that reduces HCC to < 5 cm may improve outcomes in these patients.

The potential benefit of neoadjuvant therapy in improving HCC resectability and eliminating micro-metastasis has been recognized for decades, but the study of neoadjuvant therapy in HCC has been hampered by a lack of effective therapies for this disease. Sorafenib and a related compound, regorafenib, are currently the only approved systemic therapies for the treatment of unresectable HCC. Sorafenib was recently studied in the neoadjuvant setting and demonstrated encouraging safety and feasibility, with most patients achieving an R0 resection (Bouattour *et al*, 2016). To our knowledge, neoadjuvant immunotherapy has never been studied in HCC.

The present study will investigate the safety and feasibility of neoadjuvant combination therapy with cabozantinib, an orally bioavailable tyrosine kinase inhibitor, and nivolumab, a selective PD-1 immune checkpoint inhibitor. Cabozantinib and nivolumab have each individually shown activity in HCC and are currently under investigation as monotherapies in large phase 3 trials in this disease. Additionally, the combination of cabozantinib and nivolumab (CaboNivo) was recently shown to be safe and tolerable in a phase 1 study (Apolo *et al*, 2016) and larger trials of this combination are planned in HCC, renal cell carcinoma, and bladder cancer.

It is hypothesized that CaboNivo may have a synergistic anti-tumor effect above what would be anticipated from their individual anti-tumor activity. As a PD-1 immune checkpoint inhibitor, nivolumab is thought to remove an inhibitory signal that prevents T cells from attacking cancer, thereby inducing an anti-tumor T cell response. Myeloid derived suppressor cells (MDSC) are a type of immune cell that suppresses T cell anti-tumor responses (Gabrilovich & Nagaraj, 2009). Hepatocyte growth factor (HGF) and its receptor, c-MET, promote the expansion of MDSCs in the liver, and inhibition of c-MET can specifically reduce the number of hepatic MDSCs (Yen *et al*, 2013). As a potent inhibitor of c-MET, cabozantinib is expected to reduce MDSCs in the liver and to thereby potentiate the effect of nivolumab (Goyal *et al*, 2013). These hypotheses will be explored in correlative studies with tissue obtained from this study.

4. PATIENT SELECTION

4.1 Eligibility Criteria

4.1.1 Locally advanced/borderline resectable HCC as defined by:

- Solitary tumor >5 cm

  OR
- Unilobar multifocal disease either with >3 tumors or one tumor >3 cm

  OR
- Bilobar disease with adequate future liver remnant, still technically resectable

  OR
- High risk disease features (tumor >3 cm with macrovascular invasion or tumor >3 cm with AFP>400)

- HCC may be diagnosed pathologically, or noninvasively by the American Association for the Study of Liver Diseases (AASLD) criteria or the Organ Procurement and Transplant Network (OPTN) Obligatory Diagnostic Criteria for Hepatocellular Carcinoma (HCC).

- No extrahepatic spread, no nodal disease, and no bilateral left and right branch portal vein involvement

4.1.2 Measurable disease per RECIST 1.1 as determined by the investigator

4.1.3 Age ≥ 18 years old on the day of consent

4.1.4 ECOG performance status ≤1 or Karnofsky ≥80 (**Appendix A**)

4.1.5 Patients must have normal organ and marrow function as defined below:

– White blood cell count	≥2000/ µL
– absolute neutrophil count	≥1,500/µL
– platelets	≥100,000/µL
– hemoglobin	≥9
– creatinine	Serum creatinine ≤ 1.5x ULN<br>OR
– creatinine clearance	≥40 mL/min/1.73 m^2 for patients with creatinine levels above institutional normal
– Lipase	≤ 2.0 x ULN and no radiologic or clinical evidence of pancreatitis

4.1.6 Adequate future liver remnant, and adequate liver function, based upon meeting the following laboratory criteria:

- Child-Pugh Score of A
- Total bilirubin ≤ 2 mg/dL. For subjects with known Gilbert's disease, bilirubin ≤ 3.0 mg/dL;
- Alanine aminotransferase (ALT) and aspartate aminotransferase (AST) ≤ 2.5 × ULN
- Serum albumin ≥ 2.8 g/dL
- Prothrombin time (PT)/INR or partial thromboplastin time (PTT) test ≤ 1.5 x ULN

4.1.7 Patients with chronic or acute HBV infection [as characterized by positive hepatitis B surface antigen (HBsAg) and/or hepatitis B core antibodies (anti-HBcAb) with detectable HBV DNA (≥10 IU/ml)] must be treated with effective antiviral therapy, as per institutional practices, prior to enrollment and for the duration of the study therapy. Patients who test positive for anti-hepatitis B core (HBc) with undetectable HBV DNA (<10 IU/ml) do not require anti-viral therapy prior to enrollment however these subjects will be tested at every cycle to monitor HBV DNA levels and initiate antiviral therapy if HBV DNA is detected (≥10 IU/ml).

4.1.8 Women of childbearing potential (WOCBP) must have a negative urine or serum pregnancy test for the patient to be eligible for trial enrolment.

4.1.9 WOCBP must be willing to use either two adequate barrier methods or a barrier method plus a hormonal method of contraception to prevent pregnancy, or to abstain from heterosexual activity (complete abstinence) throughout the study, starting with visit 1 through 5 months after the last dose of study therapy. Approved contraceptive methods include, for example; intrauterine device, diaphragm with spermicide, cervical cap with spermicide, male condoms, female condoms with spermicide, or oral contraceptives. Spermicides alone are not an acceptable method of contraception. Should a woman become pregnant or suspect she is pregnant while she or her partner is participating in this study, she should inform her treating physician immediately.

4.1.10 Male patients must agree to use an adequate method of contraception, or to abstain from heterosexual activity (complete abstinence), starting with the first dose of study drug through 7 months after the last dose of study therapy.

4.1.11 Ability to understand and the willingness to sign a written informed consent document.

4.2 Exclusion Criteria

4.2.1 Fibrolamellar carcinoma or mixed HCC.

4.2.2 Receiving, or previously received, any systemic chemotherapy, or investigational agent for HCC.

- Note: Prior surgical resection with recurrence, or palliative local therapy (including TACE, Y-90 Resin Microspheres, etc.) would not exclude trial participation, but must have been performed at least 30 days prior to enrollment.

4.2.3 Concomitant anticoagulation at therapeutic doses with oral anticoagulants (eg, warfarin, direct thrombin and Factor Xa inhibitors) or platelet inhibitors (eg, clopidogrel);

- Note: Low-dose aspirin for cardioprotection (per local applicable guidelines), low-dose warfarin (< 1 mg/day), and low dose, low molecular weight heparins (LMWH) are permitted. Anticoagulation with therapeutic doses of LMWH is allowed in subjects without radiographic evidence of brain metastasis, who are on a stable dose of LMWH for at least 12 weeks before randomization, and who have had no complications from a thromboembolic event or the anticoagulation regimen.

4.2.4 The subject has any of the following risks of bleeding:

- clinically-significant GI bleeding within 6 months before enrollment;
- hemoptysis of ≥ 0.5 teaspoon (2.5ml) of red blood within 3 months enrollment;
- any other signs indicative of pulmonary hemorrhage within 3 months before enrollment.
- Radiographic evidence of cavitating pulmonary lesion(s).

4.2.5 Has a known history of Human Immunodeficiency Virus (HIV)/AIDS

4.2.6 Has active co-infection with both HBV and HCV (detectable HCV RNA plus positive HBV surface antigen or HBV DNA)

4.2.7 Has active co-infection with HBV and HDV.

4.2.8 Has a diagnosis of immunodeficiency, or is receiving systemic steroid therapy above physiologic replacement dose, or any other form of immunosuppressive therapy.

4.2.9 Has active autoimmune disease that has required systemic treatment in the past 2 years (i.e. with use of disease modifying agents, corticosteroids or immunosuppressive drugs). Replacement therapy (eg., thyroxine, insulin, or physiologic corticosteroid replacement therapy for adrenal or pituitary insufficiency, etc.) is not considered a form of systemic treatment. Any patient bearing an allograft is not eligible.

4.2.10 Has a known additional malignancy that is expected to require active treatment within two years, or is likely to be life-limiting in the opinion of the treating investigator. Superficial bladder cancer, non-melanoma skin cancers, or low grade prostate cancer not requiring therapy would not exclude participation in this trial.

4.2.11 Uncontrolled intercurrent illness including, but not limited to: Symptomatic congestive heart failure, unstable angina pectoris, cardiac arrhythmia, or psychiatric illness/social situations that would limit compliance with study requirements

4.2.12 Corrected QT interval calculated by the Fridericia formula (QTcF) > 500 ms within 7 days before starting study treatment

- Note: If the QTcF is > 500 ms in first ECG, a total of 3 ECGs should be performed. If the average of these 3 consecutive results for QTcF is ≤ 500 ms, the subject meets eligibility in this regard.

4.2.13 Poorly controlled hypertension (SBP >150 or DBP >90 despite antihypertensive therapy).

4.2.14 Pregnant or lactating females

4.2.15 Gastrointestinal (GI) disorders including those associated with a high risk of perforation or fistula formation: Tumors invading the GI tract, active peptic ulcer disease, inflammatory bowel disease, (eg, Crohn's disease), diverticulitis, cholecystitis, symptomatic cholangitis or appendicitis, acute pancreatitis or acute obstruction of the pancreatic duct or common bile duct, or gastric outlet obstruction, abdominal fistula, GI perforation, bowel obstruction, intra-abdominal abscess within 6 months before randomization.

4.2.16 Any of the following within 6 months before the enrollment:
- unstable angina pectoris;
- clinically-significant cardiac arrhythmias;
- stroke (including transient ischemic attack (TIA), or other ischemic event);
- myocardial infarction;
- thromboembolic event requiring therapeutic anticoagulation (Note: subjects with a venous filter (eg, vena cava filter) are not eligible for this study).

4.2.17 Major surgery within 2 months before enrollment. Complete healing from major surgery must have occurred 1 month before enrollment. Complete healing from minor surgery (eg, simple excision, tooth extraction) must have occurred at least 7 days before enrollment. Subjects with clinically relevant complications from prior surgery are not eligible.

4.2.18 Moderate or severe ascites.

4.2.19 Subjects with untreated or incompletely treated varices with bleeding or high risk for bleeding. Subjects treated with adequate endoscopic therapy (according to institutional standards) without any episodes of recurrent GI bleeding requiring transfusion or hospitalization for at least 6 months prior to enrollment are eligible.

4.2.20 Inability to swallow intact tablets.

4.2.21 Previously identified allergy or hypersensitivity to any component of the study treatment formulations.

4.2.22 The subject requires chronic concomitant treatment with strong CYP3A4 inducers (eg, phenytoin, carbamazepine, rifampin, rifabutin, rifapentine, phenobarbital, and St. John's Wort).

4.3 Inclusion of Women and Minorities

Both men and women of all races and ethnic groups are eligible for this trial.

5. TREATMENT PLAN

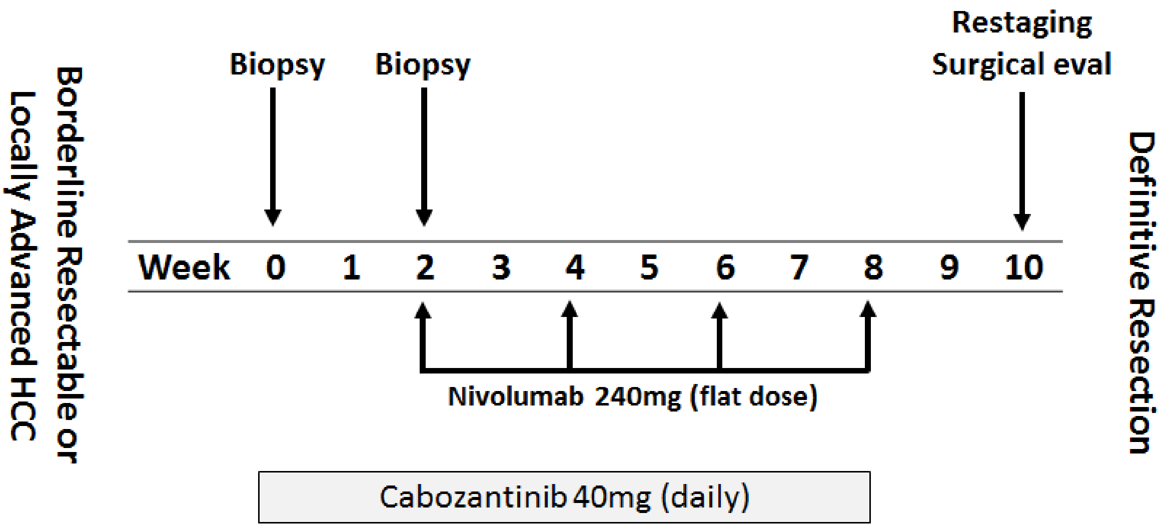

5.1 Agent Administration

Treatment will be administered on an outpatient basis over an 8 week period (see **Figure 1**, **Schema**). Dosing delays and dose modifications are described in **Section 6**. After receiving 8 weeks of therapy, patients will be evaluated for a potentially curative hepatic resection. If eligible for surgery, they will undergo definitive resection at least 28 days after the last dose of cabozantinib therapy (see **Study Calendar, Section 7** for surgery windows). Following surgery (or if they do not receive surgical resection), patients will resume standard of care therapy at the discretion of the treating oncologist.

Table 3: Study Regimen

Cabozantinib	No prophylactic pre-medications	40 mg	oral daily
nivolumab	No prophylactic pre-medications unless indicated by previous experience in an individual subject	240mg (flat dose)	IV over 60 minutes

Infusion times are approximate (+/- 10 min) and may need to be adjusted based on subject tolerability.

Subjects will receive cabozantinib orally at a (starting) dose of 40 mg once daily on day 0 and continue treatment for the entire 8 week treatment period.

Cabozantinib must be taken on an empty stomach. Subjects must be instructed not to eat for at least 2 hours before and at least 1 hour after taking cabozantinib. Subjects should be instructed to take their cabozantinib dose at approximately the same time every day. If a subject misses a dose, the dose may be taken later only if it is within 12 hours of when the missed dose should have been taken. The missed dose should not be made up if it is within 12 hours of the next scheduled dose.

If vomiting occurs shortly after the cabozantinib tablets are swallowed, the dose should only be replaced if all of the intact tablets can be seen and counted. Should any patient enrolled on the study miss a scheduled dose for whatever reason (e.g., as a result of forgetting to take the tablets or vomiting), the patient will be allowed to take the scheduled dose up to a maximum of 12 hours after that scheduled dose time. If greater than 12 hours after the scheduled dose time, the missed dose is not to be taken and the patient should take their allotted dose at the next scheduled time.

Cabozantinib tablets should be swallowed whole with at least 8 ounces of water. The tablets should not be crushed. Grapefruit, grapefruit juice, Seville oranges and their products should be avoided by subjects taking cabozantinib.

A patient handout with drug information and a wallet card is in **Appendix B**. A patient medication diary is found in **Appendix C**.

5.2 General Concomitant Medication and Supportive Care Guidelines

5.2.1 Nivolumab is a humanized monoclonal Ab. Since nivolumab contains only human immunoglobulin protein sequences, it is unlikely to be immunogenic and induce infusion or hypersensitivity reactions. However, if such a reaction were to occur, it might manifest with fever, chills, rigors, headache, rash, pruritis, arthralgias, hypo- or hypertension, bronchospasm, or other symptoms.

General treatment recommendations are provided below and may be modified based on local treatment standards and guidelines as appropriate:

- **For Grade 1 symptoms**: *(Mild reaction; infusion interruption not indicated; intervention not indicated)*

  Remain at bedside and monitor subject until recovery from symptoms. The following prophylactic premedications are recommended for future infusions: diphenhydramine 50 mg (or equivalent) and/or paracetamol 325 to 1000 mg (acetaminophen) at least 30 minutes before additional nivolumab administrations.

- **For Grade 2 symptoms**: *(Moderate reaction requires therapy or infusion interruption but responds promptly to symptomatic treatment [e.g, antihistamines, non-steroidal anti inflammatory drugs, narcotics, corticosteroids, bronchodilators, IV fluids]; prophylactic medications indicated for 24 hours).*

  Stop the nivolumab infusion, begin an IV infusion of normal saline, and treat the subject with diphenhydramine 50 mg IV (or equivalent) and/or paracetamol 325 to 1000 mg (acetaminophen); remain at bedside and monitor subject until resolution of symptoms. Corticosteroid or bronchodilator therapy may also be administered as appropriate. If the infusion is interrupted, then restart the infusion at 50% of the original infusion rate when symptoms resolve; if no further complications ensue after 30 minutes, the rate may be increased to 100% of the original infusion rate. Monitor subject closely. If symptoms recur then no further nivolumab will be administered at

that visit. Administer diphenhydramine 50 mg IV, and remain at bedside and monitor the subject until resolution of symptoms. The amount of study drug infused must be recorded on the electronic case report form (eCRF). The following prophylactic premedications are recommended for future infusions: diphenhydramine 50 mg (or equivalent) and/or paracetamol 325 to 1000 mg (acetaminophen) should be administered at least 30 minutes before additional nivolumab administrations. If necessary, corticosteroids (recommended dose: up to 25 mg of IV hydrocortisone or equivalent) may be used.

- **For Grade 3 or Grade 4 symptoms**: *(Severe reaction, Grade 3: prolonged [ie, not rapidly responsive to symptomatic medication and/or brief interruption of infusion]; recurrence of symptoms following initial improvement; hospitalization indicated for other clinical sequelae [eg, renal impairment, pulmonary infiltrates]). Grade 4: (life threatening; pressor or ventilatory support indicated).*

  Immediately discontinue infusion of nivolumab. Begin an IV infusion of normal saline, and treat the subject as follows. Recommend bronchodilators, epinephrine 0.2 to 1 mg of a 1:1,000 solution for subcutaneous administration or 0.1 to 0.25 mg of a 1:10,000 solution injected slowly for IV administration, and/or diphenhydramine 50 mg IV with methylprednisolone 100 mg IV (or equivalent), as needed. Subject should be monitored until the investigator is comfortable that the symptoms will not recur. Nivolumab will be permanently discontinued. Investigators should follow their institutional guidelines for the treatment of anaphylaxis. Remain at bedside and monitor subject until recovery from symptoms. In the case of late-occurring hypersensitivity symptoms (eg, appearance of a localized or generalized pruritis within 1 week after treatment), symptomatic treatment may be given (eg, oral antihistamine, or corticosteroids).

5.2.2 Nivolumab-Related Adverse Events

Blocking PD-1 function may permit the emergence of auto-reactive T cells and resultant clinical autoimmunity. Rash/pruritus, diarrhea/colitis, pneumonitis, hepatitis, and hypothyroidism were drug-related, presumptive autoimmune events noted in previous nivolumab studies.

For the purposes of this study, a nivolumab-related AE is defined as an AE of unknown etiology, associated with drug exposure and is consistent with an immune phenomenon. Efforts should be made to rule out neoplastic, infectious, metabolic, toxin or other etiologic causes. Serological, immunological, and histological (biopsy) data should be used to support the diagnosis of an immune-mediated toxicity. Identification and treatment of nivolumab-related AEs can be found in **Appendix D**. Additional guidance can be found in the nivolumab Investigator's Brochures (IB).

5.3 Prohibited and Restricted Therapies

Patients may NOT use any of the following agents during the study:

- Treatments aside from than those described in this treatment protocol, with the intent to treat the subject's malignancy including:
  - Systemic anticancer treatments other than nivolumab or cabozantinib
  - Localized anticancer treatments (such as surgery, ablation, or embolization)
  - Palliative external radiation
  - Investigational or commercial agents

- Therapeutic doses of oral anticoagulants (eg, warfarin or other coumarin-related agents, direct thrombin or direct FXa inhibitors, or antiplatelet agents such as clopidogrel, or chronic use of aspirin above low dose levels for cardioprotection per local applicable guidelines).

<u>The following therapies should be avoided if possible, while the subject is on study:</u>

- Systemically active steroids can be used but should be reported to the Principal Investigator. In general, steroid treatment should be reduced to physiologic doses prior to treatment with nivolumab.

- Erythropoietic stimulating agents (eg, epoetin alfa and darbepoetin alfa) should not be used based on a report of increased risk of tumor recurrence and/or progression associated with erythropoietin (Wright 2007).

- Chronic co-administration of cabozantinib with strong inducers of the CYP3A4 family (eg, phenytoin, carbamazepine, rifampin, rifabutin, rifapentine, phenobarbital, and St. John's Wort) may significantly decrease cabozantinib concentrations and should be avoided. Selection of alternate concomitant medications with no or minimal CYP3A4 enzyme induction potential is recommended.

- Caution must be used when discontinuing treatment with a strong CYP3A4 inducer in a subject who has been concurrently receiving a stable dose of cabozantinib, because this could significantly increase the exposure to cabozantinib.

- Co-administration of cabozantinib with strong inhibitors of the CYP3A4 family (eg, ketoconazole, itraconazole, clarithromycin, indinavir, nefazodone, nelfinavir, and ritonavir) may increase cabozantinib concentrations and should be avoided. Grapefruit and Seville oranges may also increase plasma concentrations of cabozantinib and should be avoided.

5.3.1 Potential Drug Interactions

- Cytochrome P450 (CYP): Data from a clinical drug interaction study (Study XL184-008) show that clinically relevant steady-state concentrations of cabozantinib appear to have no marked effect on the area under the plasma drug concentration time curve (AUC) of co-administered rosiglitazone, a CYP2C8 substrate. Therefore, cabozantinib is not anticipated to markedly inhibit CYP2C8 in the clinic, and by inference, is not anticipated to markedly inhibit other CYP450 isozymes that have lower [I]/Ki values compared with CYP2C8 (ie, CYP2C9, CYP2C19, CYP2D6, CYP1A2, and CYP3A4). In vitro data indicate that cabozantinib is unlikely to induce cytochrome P450 enzymes, except for possible induction of CYP1A1 at high cabozantinib concentrations (30 µM).

- Cabozantinib is a CYP3A4 substrate and a weak substrate for CYP2C9 (but not a CYP2D6, CYP2C8, CYP2C19, CYP2B6, or CYP1A2 substrate), based on data from in vitro studies. Results from a clinical pharmacology study, (Study XL184-006), showed that concurrent administration of cabozantinib with the strong CYP3A4 inducer, rifampin, resulted in an approximately 77% reduction in cabozantinib exposure (AUC values) after a single dose of cabozantinib in healthy volunteers. Chronic co-administration of cabozantinib with strong inducers of the CYP3A4 family (eg, phenytoin, carbamazepine, rifampin, rifabutin, rifapentine, phenobarbital, and St. John's Wort) may significantly decrease cabozantinib concentrations. The chronic use of strong CYP3A4 inducers should be avoided. Other drugs that induce CYP3A4 should be used with caution because these drugs have the potential to decrease exposure (AUC) to cabozantinib. Selection of alternate concomitant medications with no or minimal CYP3A4 enzyme induction potential is recommended.

- Results from a clinical pharmacology study, (Study XL184-007), showed that concurrent administration of cabozantinib with the strong CYP3A4 inhibitor, ketoconazole, resulted in a 38% increase in the cabozantinib exposure (AUC values) after a single dose of cabozantinib in healthy volunteers. Co-administration of cabozantinib with strong inhibitors of the CYP3A4 family (eg, ketoconazole, itraconazole, clarithromycin, indinavir, nefazodone, nelfinavir, and ritonavir) may increase cabozantinib concentrations. Grapefruit and Seville oranges may also increase plasma concentrations of cabozantinib and should be avoided. Strong CYP3A4 inhibitors should be avoided and other drugs that inhibit CYP3A4 should be used with caution because these drugs have the potential to increase exposure (AUC) to cabozantinib. Selection of alternate concomitant medications with no or minimal CYP3A4 enzyme inhibition potential is recommended.

- In addition, cimetidine should be avoided because of its potential to interfere with CYP3A4 mediated metabolism of cabozantinib.

- Please refer to the drug interaction tables at the following websites for lists of substrates, inducers, and inhibitors of selected CYP450 isozyme pathways:
    - Http://medicine.iupui.edu/clinpharm/ddis/table.aspx
    - http://www.fda.gov/Drugs/DevelopmentApprovalProcess/DevelopmentResources/DrugInteractionsLabeling/ucm080499.htm

- Protein Binding: Cabozantinib is highly bound (≥ 99.7%) to human plasma proteins. Therefore, highly protein bound drugs should be used with caution with cabozantinib because there is a potential displacement interaction that could increase free concentrations of cabozantinib and/or a co-administered highly protein-bound drug (and a corresponding increase in pharmacologic effect).

- Other Interactions:

  - Food may increase exposure levels of cabozantinib by 57%, fasting recommendations should be followed. Subjects should fast (with the exception of water) for at least 2 hours after eating the evening meal before taking their dose of cabozantinib. After the 2-hour fast and before going to bed, subjects are to take cabozantinib with a full glass of water (minimum of 8 oz or 240 mL) with no more food intake for one hour post-dose.
  - In vitro data suggest that cabozantinib is unlikely to be a substrate for P-glycoprotein, but it does appear to have the potential to inhibit the P-glycoprotein transport activity. Therefore, cabozantinib may have the potential to increase plasma concentrations of co-administered substrates of P-glycoprotein.
  - Cabozantinib was shown to be a substrate of drug transporter MRP2 in an in vitro assay. Administration of MRP2 inhibitors to subjects may result in increases in cabozantinib plasma concentrations.
  - Additional details regarding potential drug interactions with cabozantinib can be found in the investigator brochure.

5.4 Definition of an Overdose for this Protocol

An overdose is defined as the accidental or intentional administration of any dose of a product that is considered both excessive and medically important. All occurrences of overdose must be reported as SAEs.

5.5 WOCBP, Contraception, Use in Pregnancy, Use in Nursing:

5.5.1 WOCBP

A WOCBP is defined as any female who has experienced menarche and who has not undergone surgical sterilization (hysterectomy or bilateral oophorectomy) and is not postmenopausal. Menopause is defined as 12 months of amenorrhea in a woman over age 45 years in the absence of other biological or physiological causes. In addition, women under the age of 55 years must have a serum follicle stimulating hormone (FSH) level > 40mIU/mL to confirm menopause.

Women treated with hormone replacement therapy (HRT) are likely to have artificially suppressed FSH levels and may require a washout period in order to obtain a physiologic FSH level. The duration of the washout period is a function of the type of HRT used. The duration of the washout periods below are suggested guidelines and the investigators should use their judgment in checking serum FSH levels. If the serum FSH level is >40 mIU/ml at any time during the washout period, the woman can be considered postmenopausal:

- 1 week minimum for vaginal hormonal products (rings, creams, gels)
- 4 week minimum for transdermal products
- 8 week minimum for oral products

5.5.2 Contraception

The investigational agents used in this protocol may have adverse effects on a fetus in utero. Furthermore, it is not known if the investigational agents have transient adverse effects on the composition of sperm. Non-pregnant, non-breast-feeding women may be enrolled if they are considered highly unlikely to conceive. Highly unlikely to conceive is defined as 1) surgically sterilized, or 2) postmenopausal (a woman who is ≥45 years of age and has not had menses for greater than 12 months will be considered postmenopausal), or 3) amenorrheaic for <12 months without a hysterectomy and oophorectomy and with a documented FSH value in the postmenopausal range (>30 IU/L), or 4) not heterosexually active for the duration of the study, or 5) heterosexually active and willing to use 2 methods of birth control. The 2 birth control methods can be 2 barrier methods or a barrier method plus a hormonal method to prevent pregnancy, used throughout the study starting with enrollment through 5 months after the last dose of study medication. Male subjects enrolled in this study must also agree to use an adequate method of contraception starting with enrollment through 7 months after the last dose of study drug.

Investigators shall counsel WOCBP and male subjects who are sexually active with WOCBP on the importance of pregnancy prevention and the implications of an unexpected pregnancy. Investigators shall advise WOCBP and male subjects who are sexually active with WOCBP on the use of highly effective methods of contraception. Highly effective methods of contraception have a failure rate of < 1% per year when used consistently and correctly.

HIGHLY EFFECTIVE METHODS OF CONTRACEPTION

- Male condoms with spermicide
- Hormonal methods of contraception including combined oral contraceptive pills, vaginal ring, injectables, implants, and intrauterine devices (IUDs) such as Mirena by WOCBP subject or male subject's WOCBP partner.
- Nonhormonal IUDs, such as ParaGard
- Tubal ligation
- Vasectomy
- Complete abstinence

Complete abstinence is defined as complete avoidance of heterosexual intercourse and is an acceptable form of contraception for all study drugs. Abstinence is only acceptable when this is in line with the preferred and usual lifestyle of the subject. Periodic abstinence (e.g., calendar, ovulation, symptothermal, profession of abstinence for entry into a clinical trial, post-ovulation methods) and withdrawal are not acceptable methods of contraception. Subjects who choose complete abstinence are not required to use a second method of contraception, but female subjects must continue to have pregnancy tests. Acceptable

alternate methods of highly effective contraception must be discussed in the event that the subject chooses to forego complete abstinence.

LESS EFFECTIVE METHODS OF CONTRACEPTION

- Diaphragm with spermicide
- Cervical cap with spermicide
- Vaginal sponge
- Male condom without spermicide*
- Progestin only pills by WOCBP subject or male subject's WOCBP partner
- Female condom*

*A male and female condom must not be used together

Subjects should be informed that taking the study medication may involve unknown risks to the fetus (unborn baby) if pregnancy were to occur during the study. In order to participate in the study they must adhere to the contraception requirement for the duration of the study treatment and for a period of time after the completion of treatment as described above. If there is any question that a subject will not reliably comply with the requirements for contraception, that subject should not be entered into the study.

5.5.3 Use in Pregnancy

The investigational agents used in this protocol may have adverse effects on a fetus; therefore, women with a positive pregnancy test at screening will not be eligible for enrollment. If a subject inadvertently becomes pregnant while on treatment, the subject will immediately be removed from the study treatment. The study team will contact the subject at least monthly and document the subject's status until the pregnancy has been completed or terminated.

The investigator must immediately notify the IND Sponsor and BMS of any pregnancy using the Pregnancy Surveillance Form within 24 hours of notification and in accordance with the SAE reporting procedures described in **Section 7.5**. Any pregnancy that occurs in a female partner of a male study participant should also be reported to the IND Sponsor and BMS.

Protocol required procedures for study treatment discontinuation and follow-up must be performed on the subject unless contraindicated by pregnancy (e.g., x-ray studies). Other appropriate pregnancy follow-up procedures should be considered if indicated.

Follow-up information regarding the course of the pregnancy, including perinatal and neonatal outcome and where applicable, offspring information must be reported on the Pregnancy Surveillance Form.

5.5.4 Use in Nursing Women

Since many drugs are excreted in human milk, and because of the potential for serious adverse reactions in the nursing infant, subjects who are breast-feeding are not eligible for enrollment.

5.6 Unacceptable Toxicities

Unacceptable toxicities are defined as:

- Treatment-related ≥ grade 4 AEs, or
- Treatment-related grade 3 AEs
- All grade 3 or 4 ocular adverse events
- ≥ grade 2 eye pain or reduction of visual acuity that does not respond to topical therapy and does not improve to ≤ grade 1 severity within 2 weeks of starting therapy, or requires systemic therapy
- Delay in planned surgery for HCC of at least 60 days beyond the planned date for surgical evaluation, due to a treatment-related adverse event

Exceptions include:

- Grade 4 endocrinopathy adverse events, such as adrenal insufficiency, ACTH deficiency, hyper- or hypothyroidism, or glucose intolerance/ hyperglycemia, or hypophysitis, which resolve or are adequately controlled with physiologic hormone replacement (corticosteroids, thyroid hormones) or glucose-controlling agents, respectively, may not require discontinuation after discussion with and approval from the Principal Investigator.
- Grade 3 endocrinopathies adequately controlled with only physiologic hormone replacement.
- Isolated electrolyte imbalances/abnormalities or other laboratory abnormalities that are not associated with clinical sequelae and are corrected with supplementation/appropriate management within 72 hours of their onset.
- Grade ≥ 3 drug-related amylase or lipase abnormality that is not associated with symptoms or clinical manifestations of pancreatitis
- Grade 3 dermatologic AEs that are considered mild in severity but only considered grade 3 because of >30% body surface involvement
- Diarrhea or vomiting that resolves to < grade 3 within 24 hours of intervention
- Fatigue, any grade
- Delay of surgery that is not due to a treatment-related adverse event (for example, if the patient declines surgery or the tumor is not resectable).

5.7 Criteria for Removal from Treatment

A subject must be discontinued from the trial for any of the following reasons:

- The patient or legal representative (such as a parent or legal guardian) withdraws consent for follow-up,

- Termination of the study, and

- Patient is lost to follow up

A subject must be discontinued from treatment (but may continue to be monitored in the post-treatment follow-up portion of the trial) for any of the following reasons:

- Development of distant metastatic disease,
- Treatment of both cabozantinib and nivolumab is delayed or interrupted for > 3 weeks (either continuous or cumulatively) the subject must permanently discontinue study treatment and move directly to surgery.
- The patient or legal representative (such as a parent or legal guardian) withdraws consent for treatment but not follow-up,
- Intercurrent illness that prevents further administration of treatment,
- Unacceptable adverse event(s) (see **Section 5.6**),
- If in the opinion of the Investigator, a change or temporal or permanent discontinuation of therapy would be in the best interest of the patient,
- Noncompliance with trial treatment or procedure requirements,
- Patient becomes pregnant. All women of child bearing potential should be instructed to contact the investigator immediately if they suspect they might be pregnant (e.g., missed or late menstrual period) at any time during study participation, and
- Patient is lost to follow-up

5.8 End of Treatment (EOT) Visit

All subjects will return to the study site 30 days (± 7 days) after the final study treatment (i.e., completion of the final course or upon early discontinuation) for an EOT evaluation. Procedures and assessments performed at these visits and beyond should follow the respective guidelines described in **Sections 5.9 and 9.0** as appropriate. The patient will be monitored for adverse events up to the mandatory EOT visit or to resolution of toxicity to Grade 0-1, whichever occurs later. In general, the EOT visit should occur prior to surgical resection; if patients proceed with surgery prior to the EOT visit then surgical-related AEs will not be collected. All attempts will be made to obtain disease-free and overall survival data on each patient after the EOT visit.

5.9 Duration of Follow Up

Treated subjects will begin the follow-up period after they complete the EOT visit. All subjects should continue to be monitored for disease status by radiologic imaging at the discretion of the treating oncologist or surgeon. All relevant radiographic imaging performed up to the time of recurrence, progression, or 5 years after study closure (whichever comes first), should be collected by the treatment team.

Subjects who complete or discontinue from treatment will be contacted (by phone or email) every three months (+/- 2 weeks) after completion of the EOT visit for up to 5 years or study closure (whichever comes first) to monitor overall survival and disease free survival. Information of other cancer therapies after discontinuation from the study treatment will

also be collected. In addition, subjects will also be contacted at 100 days (+14 day reporting window) from the last dose of nivolumab to monitor drug toxicity. SAEs must be collected up to 100 days after last dose of nivolumab, regardless of subsequent anti-cancer therapy..

Subjects who are discontinued from the study treatment due to an unacceptable drug-related AE will be monitored for safety until the resolution of the AE to ≤ grade 1 or stabilization or until initiation of a new therapy for their cancer, whichever occurs first.

6. DOSING DELAYS/DOSE MODIFICATIONS

6.1 Dosing Criteria and General Dose Modifications for Cabozantinib

- As a general approach all AEs should be managed with supportive care at the earliest signs of toxicity. Should this be ineffective, dose reductions or interruptions should be considered to prevent worsening of toxicity.

- In order to standardize the management of AEs, suggested treatment management algorithms for adverse events of particular concern are included in **Appendix D**. Additional AE treatment management algorithms included in the cabozantinib IB might be considered for individual cases.

- One dose reduction of cabozantinib to 50% of the baseline dose level (i.e. to 20 mg a day) will be permitted. Doses of cabozantinib may be modified at any time during study treatment. Cabozantinib dose modification criteria for study treatment are shown below in **Table 4**.

Table 4: Recommended cabozantinib dose modification table

Toxicity Criteria for Cabozantinib	Recommended Guidelines for Management
Grade 1 AEs	Continue study treatment if AE is tolerated
Grade 2 AEs which are intolerable or cannot be adequately managed	At the discretion of the investigator, study treatment may be reduced or interrupted. It is recommended that dose interruptions be as brief as possible
Grade 3 AEs (except for clinically non-relevant laboratory abnormalities)	Study treatment should be interrupted unless the toxicity can be easily managed with a dose reduction and optimal medical care.
Grade 4 AEs	Subjects should have their treatment interrupted. Discontinue cabozantinib treatment unless the following criteria are met:<br>1) In the opinion of the investigator the patient is deriving clinical benefit from the therapy and it is in the patient's interest to continue cabozantinib therapy<br>2) Toxicity can be managed with a dose reduction<br>3) AE is not a unacceptable toxicity (See section 5.6)

- Cabozantinib treatment should be discontinued if a dose of 20mg oral daily is not tolerated.

- Dose modifications may also occur in the setting of lower grade toxicity than defined, if the investigator feels it is in the interest of a subject's safety, or if the dose is not tolerated.

- Re-escalation of cabozantinib may be allowed if there is resolution of the AE that lead to the dose reduction, and the investigator believes that dose escalation is in the patient's interest. The maximum treatment dose of cabozantinib is 40 mg oral daily in this protocol.

If treatment is delayed > 2 weeks or permanently discontinued, the investigator should be contacted to determine if it is in the patient's best interest to discontinue therapy and proceed directly to surgical evaluation. Patients who discontinue cabozantinib due to intolerance or treatment-related adverse events may continue nivolumab monotherapy on protocol if the investigator feels that it is in the interest of the patient.

6.2 Dosing Criteria for Nivolumab

The dose of nivolumab cannot be modified. It is recommended that the dosing of nivolumab be delayed for any of the criteria:

- Any Grade ≥ 2 non-skin, drug-related adverse event, with the following exception:
  - Grade 2 drug-related fatigue does not require a treatment delay.
  - Grade 2 AST, ALT or total bilirubin abnormalities due to nivolumab-related hepatitis
  - Any Grade 3 skin, drug-related adverse event

- Any Grade 3 drug-related laboratory abnormality, with the following exceptions for lymphopenia, and asymptomatic amylase or lipase:
  - Grade 3 lymphopenia does not require dose delay
  - Any Grade ≥ 3 drug-related amylase or lipase abnormality that is not associated with symptoms or clinical manifestations of pancreatitis does not require dose delay.
- Any adverse event, laboratory abnormality, or intercurrent illness which, in the judgment of the investigator, warrants delaying the dose of study medication.

Subjects who require delay of nivolumab should be re-evaluated weekly or more frequently if clinically indicated and resume nivolumab dosing when re-treatment criteria are met.

In order to standardize the management of AEs, suggested treatment management algorithms for adverse events of particular concern are included in **Appendix D**. Additional AE treatment management algorithms included in the nivolumab IB might be considered for individual cases.

Subjects may resume treatment with nivolumab when the treatment-related AE(s) resolve to grade ≤1 or baseline value, with the following exceptions:

- Subjects may resume treatment in the presence of grade 2 fatigue.
- Subjects who have not experienced a Grade 3 drug-related skin AE may resume treatment in the presence of Grade 2 skin adverse event
- Treatment-related pulmonary toxicity or suspected colitis must have resolved to baseline before treatment is resumed.
- Treatment-related endocrinopathies adequately controlled with only physiologic hormone replacement may resume treatment, which include grade 2 hyperglycemia, hypothyroidism and thyroiditis.

6.2.1 Nivolumab treatment should be permanently discontinued for the following:

Any Grade 2 drug-related uveitis, eye pain or blurred vision that does not respond to topical therapy and does not improve to Grade 1 severity within the re-treatment period OR requires systemic treatment

- Any Grade 3 non-skin, drug-related adverse event lasting > 7 days or recurs, with the following exceptions for laboratory abnormalities, drug-related uveitis, pneumonitis, bronchospasm, hypersensitivity reactions, infusion reactions, and endocrinopathies:
- Grade 3 drug-related uveitis, pneumonitis, bronchospasm, hypersensitivity reaction, or infusion reaction of any duration requires discontinuation
- Grade 3 drug-related endocrinopathies adequately controlled with only physiologic hormone replacement do not require discontinuation
- Grade 3 drug-related laboratory abnormalities do not require treatment discontinuation except:
  - Grade 3 drug-related thrombocytopenia > 7 days or associated with bleeding requires discontinuation
  - Concurrent AST or ALT > 3 x ULN AND total bilirubin > 2x ULN
  - Grade ≥ 3 drug-related AST, ALT or total bilirubin requires discontinuation*
  - *In most cases of Grade 3 AST or ALT elevation, study drug(s) will be permanently discontinued.

If nivolumab treatment is delayed > 2 weeks or permanently discontinued, the investigator should be contacted to determine if it is in the patient's best interest to discontinue therapy and proceed to surgical evaluation. Patients who discontinue nivolumab due to intolerance or treatment-related adverse events may continue cabozantinib monotherapy on protocol if the investigator feels that it is in the interest of the patient.

7. ADVERSE EVENTS: LIST AND REPORTING REQUIREMENTS

This study will use the descriptions and grading scales found in the revised CTCAE version 4.03 for AE reporting that can be found at:

http://ctep.cancer.gov/protocolDevelopment/electronic_applications/ctc.htm.

Information about all adverse events, whether volunteered by the subject, discovered by investigator questioning, or detected through physical examination, laboratory test or other means, will be collected, recorded, and followed as appropriate.

The PI has the primary responsibility for continuous internal monitoring for safety, protocol compliance, and identification, grading, coding, and required reporting of all anticipated and unanticipated adverse events and protocol problems. Although this responsibility is usually shared among the PI, research nurse, and data manager, the PI is ultimately responsible for grading and attribution of all events.

7.1 Definitions

7.1.1 Adverse Events (AEs)

An adverse event is defined as any undesirable sign, symptom or medical condition occurring after starting the study drug (or therapy) even if the event is not considered to be related to the study treatment. An undesirable medical condition can be symptoms (e.g., nausea, chest pain), signs (e.g., tachycardia, enlarged liver) or the abnormal results of an investigation (e.g., laboratory findings, electrocardiogram). Medical conditions/diseases present before starting the study treatment are only considered adverse events if they worsen after starting the study treatment (any procedures specified in the protocol. Progression of the disease being studied will not be recorded as an adverse event. Abnormal laboratory values or test results constitute adverse events only if they induce clinical signs or symptoms or require therapy.

Laboratory abnormalities present at the screening visit will be recorded as pre-treatment signs and symptoms. After study treatment administration, all grade 3 and 4 clinical laboratory results that represent an increase in severity from baseline will be reported as adverse events. A grade 1 or 2 clinical laboratory abnormality should be reported as an adverse event only if it is considered clinically significant by the investigator (induce clinical signs or symptoms or require therapy).

7.1.2 Serious Adverse Event (SAE)

A serious adverse event is an undesirable sign, symptom or medical condition which:

- Results in death
- Is life threatening (defined as an event in which the subject was at risk of death at the time of the event; it does not refer to an event which hypothetically might have caused death if it were more severe)
- Requires inpatient hospitalization or causes prolongation of existing hospitalization > 24 hours (see note below for exceptions)
- Results in persistent or significant disability/incapacity
- Is a congenital anomaly/birth defect (note: reports of congenital anomalies/birth defects must also be reported on the Pregnancy Supplemental Form)
- Is an important medical event (defined as a medical event(s) that may not be immediately life-threatening or result in death or hospitalization but, based upon appropriate medical and scientific judgment, may jeopardize the subject or may require intervention [eg, medical, surgical] to prevent one of the other serious outcomes listed in the definition above. Examples of such events include, but are not limited to, intensive treatment in an emergency room or at home for allergic bronchospasm; blood dyscrasias or convulsions that do not result in hospitalization.)

- Is a new cancer (that is not a condition of the study)
- Is associated with an overdose
- Potential drug induced liver injury (DILI) is also considered an important medical event.
- Hemophagocytic lymphohistiocytosis is also considered an important medical event.
- Suspected transmission of an infectious agent (eg, pathogenic or nonpathogenic) via the study drug is an SAE.
- Is a pregnancy or pregnancy outcome of spontaneous abortion, missed abortion, benign hydatidiform mole, blighted ovum, fetal death, intrauterine death, miscarriage, or stillbirth.

Events **not** considered to be serious adverse events are hospitalizations for the:

- Admissions as per protocol for a planned medical/surgical procedure or to facilitate a procedure
- Routine health assessment requiring admission for baseline/trending of health status (eg, routine colonoscopy)
- Medical/surgical admission for purpose other than remedying ill health state and was planned prior to entry into the study. Appropriate documentation is required in these cases
- Admission encountered for another life circumstance that carries no bearing on health status and requires no medical/surgical intervention (eg, lack of housing, economic inadequacy, care-giver respite, family circumstances, administrative).
- Admissions for monitoring of treatment-related infusion reactions that do not otherwise meet the criteria for a SAE.

7.2 Relationship and Grading

The relationship between the AE and the study treatment will be determined by the principal investigator.

Yes (related): Adverse events that are at least possibly related to study treatment. The time course between the administration of study drug and the occurrence or worsening of the adverse event is consistent with a causal relationship and no other cause (concomitant drugs, therapies, complications, etc.) can be identified.

No (unrelated, not related, no relation): The time course between the administration of study drug and the occurrence or worsening of the adverse event rules out a causal relationship and another cause (concomitant drugs, therapies, complications, etc.) is suspected.

The following factors should also be considered:
- The temporal sequence from study drug administration - The event should occur after the study drug is given. The length of time from study drug exposure to event should be evaluated in the clinical context of the event.
- Underlying, concomitant, intercurrent diseases - Each report should be evaluated in the context of the natural history and course of the disease being treated and any other disease the subject may have.

- Concomitant medication - The other medications the subject is taking or the treatment the subject receives should be examined to determine whether any of them might be recognized to cause the event in question.
- Known response pattern for this class of study drug - Clinical and/or preclinical data may indicate whether a particular response is likely to be a class effect.
- Exposure to physical and/or mental stresses - The exposure to stress might induce adverse changes in the recipient and provide a logical and better explanation for the event.

The pharmacology and pharmacokinetics of the study drug - The known pharmacologic properties (absorption, distribution, metabolism, and excretion) of the study drug should be considered.

Assessment of Grade:

The investigator will make an assessment of grade for each AE and SAE reported during the study, which will be recorded in the CRF. The assessment will be based on the National Cancer Institute's CTCAE (Version 4.03) and graded as shown below:

- Grade 1: Mild; asymptomatic or mild symptoms; clinical or diagnostic observations only; intervention not indicated
- Grade 2: Moderate; minimal, local or noninvasive intervention indicated; limiting age-appropriate instrumental activities of daily living
- Grade 3: Severe or medically significant but not immediately life-threatening; hospitalization or prolongation of hospitalization indicated; disabling; limiting self-care activities of daily living
- Grade 4: Life-threatening consequences; urgent intervention indicated
- Grade 5: Death related to AE

Any AE that changes in grade during its course will be recorded in the CRF at the highest level experience by the subject.

7.3 Expectedness

<u>Unexpected adverse event:</u> An adverse event, which varies in nature, intensity or frequency from information on the investigational drug/agent provided in the Investigator's Brochure (IB), package insert or safety reports. Any adverse event that is not included in the informed consent is considered "unexpected".

<u>Expected (known) adverse event:</u> An adverse event, which has been reported in the IB. An adverse event is considered "expected", only if it is included in the informed consent document as a risk.

7.4 Handling of Expedited Safety Reports

In accordance with local regulations, the IND Sponsor will notify investigators of all SAEs that are unexpected (i.e., not previously described in the Investigator's Brochure (IB) and/or package inserts), and possibly, probably, or definitely related to cabozantinib or

nivolumab. This notification will be in the form of an expedited safety report (ESR) that is to be faxed to the investigators and the study coordinators. Upon receiving such notices, the investigator must review and retain the notice with the IB and where required by local regulations, the investigator will submit the ESR to the appropriate investigational review board (IRB). The investigator and IRB will determine if the informed consent requires revision. The investigator should also comply with the IRB procedures for reporting any other safety information.

7.5 Reporting

7.5.1 Adverse Events and Serious Adverse Events

All adverse events (both expected and unexpected) will be captured on the appropriate study-specific case report forms (CRFs). Adverse events experienced by subjects will be collected and reported from the first dose of the study drug, throughout the study, and will only be followed for 30 days (or within 7 days prior to initiation of a new anti-cancer treatment, whichever comes first) unless related to the study drug(s). Adverse events related to the study drug(s) will be monitored for resolution of toxicity to ≤ grade 1, stabilization, or determined to be irreversible by the investigator. Surgery is considered standard of care (SOC).

Report AEs to the IND Sponsor within 24 hours once identified as an unacceptable toxicity (defined in Section 5.6).

IND Sponsor: laherda@jhmi.edu

SAEs that occur from the first dose of study drug to within 100 days of the last infusion of nivolumab (or before initiation of a new antineoplastic treatment, whichever occurs first), with the exception of surgical complications should be followed and recorded.

All SAEs, regardless of causality to study drug, will be reported promptly to the IND sponsor (e-mail: laherda@jhmi.edu), BMS (e-mail: Worldwide.Safety@BMS.com), and Exelixis (drugsafety@exelixis.com or fax 650-837-7392) within 24 hours of recognition of the SAE, even if it is not felt to be drug related, using the SAE reporting form (**Appendix E**). If this falls on a weekend or holiday, an email notification is acceptable but must be followed by an SAE reporting form on the next business day.

7.5.2 Follow-up of Adverse Events and Serious Adverse Events

After the initial AE or SAE report, the investigator is required to proactively follow each subject and provide further information to the safety department in regards to the subject's condition.

All AE(s) and SAE(s) will be followed until:

- Resolution
- The condition stabilizes
- The event is otherwise explained
- The subject is lost to follow-up or dies

Once the event is resolved, the appropriate AE or SAE report page will be updated. The investigator will also ensure that the follow-up includes any supplemental information that may explain the causality of the event(s). New or updated information will be recorded on the originally completed AE or SAE report, with all changes signed and dated by the investigator or designee. The updated AE or SAE report will then be signed by the investigator and resubmitted to the IND Sponsor.

7.5.3 Overdose

An overdose is defined as the accidental or intentional administration of any dose of a product that is considered both excessive and medically important. All occurrences of overdose must be reported as SAEs.

7.5.4 Potential Drug Induced Liver Injury (DILI)

Wherever possible, timely confirmation of initial liver-related laboratory abnormalities should occur prior to the reporting of a potential DILI event. All occurrences of potential DILIs, meeting the defined criteria, must be reported as SAEs under the seriousness category checked as ‘other medically important event’. Potential drug induced liver injury is defined as:

1) ALT or AST elevation > 10 times upper limit of normal (ULN)
   AND
2) Total bilirubin > 2 times ULN, without initial findings of cholestasis (elevated serum alkaline phosphatase)
   AND
3) No other immediately apparent possible causes of AST/ALT elevation and hyperbilirubinemia, including, but not limited to, viral hepatitis, pre-existing chronic or acute liver disease, or the administration of other drug(s) known to be hepatotoxic.

7.5.5 Pregnancy Reporting

Although pregnancy and lactation are not considered AEs, it is the responsibility of investigators or their designees to report any pregnancy or lactation in a subject (spontaneously reported to them) that occurs during the trial or within 120 days after the last dose of nivolumab. This also includes the pregnancy of a male subject's female partner who has provided written consent to provide information regarding pregnancy, that occurs during the trial or within 120 days of completing the trial. All subjects who become pregnant must be followed to the completion/termination of the pregnancy. Pregnancy outcomes of spontaneous abortion, missed abortion, benign hydatidiform mole, blighted ovum, fetal death, intrauterine death, miscarriage and stillbirth must be reported as SAEs (Important Medical Events). If the pregnancy continues to term, the outcome (health of infant) must also be reported to the IND sponsor.

7.5.6 Institutional Review Board

SAEs will be reported to the IRB per institutional standards. Follow-up information will be submitted to the IRB as soon as relevant information is available.

7.5.7 Food and Drug Administration (FDA)

All reporting to the FDA will be completed by the IND Sponsor.

7.5.7.1 Expedited IND Safety Reports:

7 Calendar-Day Telephone or Fax Report:
The IND Sponsor is required to notify the FDA of any fatal or life-threatening adverse event that is unexpected and assessed by the investigator to be possibly related to the investigational agent. Such reports are to be telephoned or faxed (301-796-9849) to the FDA within 7 calendar days of first learning of the event. Follow-up information will be submitted to the FDA as soon as relevant information is available.

15 Calendar-Day Written Report:
The IND Sponsor is required to notify the FDA of any SAE that is unexpected and related to the investigational agent in a written IND Safety Report.

Written IND Safety Reports should include an Analysis of Similar Events in accordance with regulation 21 CFR § 312.32. All safety reports previously filed with the IND concerning similar events should be included in the analysis. The new report should contain comments on the significance of the new event in light of the previous, similar reports.

Written IND safety reports with Analysis of Similar Events are to be submitted to the FDA within 15 calendar days of first learning of the event. Follow-up information will be submitted to the FDA as soon as relevant information is available.

7.5.7.2 IND Annual Reports

In accordance with the regulation 21 CFR § 312.33, the IND Sponsor shall within 60 days of the anniversary date that the IND went into effect submit a brief report of the adverse events and progress of the investigation. Please refer to Code of Federal Regulations, 21 CFR § 312.33 for a list of the elements required for the annual report. All IND annual reports will be submitted to the FDA by the IND Sponsor.

7.6 Special considerations for adverse events that occur during the surgery and postoperative course

Surgical resection for HCC is a high risk procedure that requires prolonged hospitalization, has significant toxicities, and is commonly associated with complications and comorbidities. It is a standard of care, therefore, is not part of this research study. From the day of surgery throughout the postoperative course day 30, participants will be primarily followed by their primary surgeons for monitoring and managing the complications and comorbidities attributable to the surgery. Surgical AEs will not be routinely collected as part of this study protocol.

8. BIOMARKER, CORRELATIVE, AND SPECIAL STUDIES

8.1 Whole blood

Whole blood, up to 150 CCs, will be collected for PBMCs and/or plasma. Refer to the study calendar or laboratory manual.

8.2 Tumor Tissue Studies

4-6 core tumor biopsies will be collected prior to treatment initiation and after a two week lead in of cabozantinib therapy. Additional tumor tissue will be collected from surgical resection samples for patients who continue to a surgical resection. Detailed instructions for tissue collection, processing, and shipment are provided in the Laboratory Manual.

The purpose of these tumor tissue studies is to determine the effect of cabozantinib monotherapy, and combination therapy with nivolumab, on tumor infiltrating lymphocytes as well as immune activation pathways, immune suppressive pathways, and cytokine/chemokine signaling.

Table 5: Proposed Biomarker Table

Biomarker	Assay	Use (Integral, Integrated, or Exploratory)AND Purpose [b]	Tissue/Body Fluid Tested and Timing of Assay
CD8+ T effector cells and CD4+FoxP3+ T regulatory cells	IHC	To explore changes in the tumor microenvironment that may impact response to therapy.	Tumor tissue<br>Pre-treatment, week 2, and at surgical resection
PD-L1 expression on tumor and tumor infiltrating lymphocytes (TILs)	IHC	To explore changes in the tumor microenvironment that may impact response to therapy	Tumor tissue<br>Pre-treatment, week 2, and at surgical resection
MHC I expression	IHC	To explore changes in MHC I expression on cabozantinib treatment, in order to provide a mechanistic rationale for the combination	Tumor tissue<br>Pre-treatment, week 2, and at surgical resection
Immunohistochemistry (IHC) and transcriptional analysis	IHC	To explore the effect of cabozantinib on immune activation pathways, immune suppressive pathways, and cytokine/chemokine signaling	Tumor tissue<br>Pre-treatment, week 2, and at surgical resection

In addition, we will use CITE-Seq, RNA-Seq, whole exome sequencing, whole genome sequencing, T cell receptor and B cell receptor sequencing, ChIP-seq, ATAC-seq, and MBD-seq to explore the effects of therapy on tumor and tumor infiltrating immune cells.

Personalis, Inc (vendor) will perform nucleic acid extraction and sequencing analysis on the tumor specimens that will provide information regarding tumor mutation burden, neontigens, immune pathways expression, ant T cell receptor repertoire. This information will provide data in order to determine the effect of immunotherapies in hepatocellular carcinoma and potentially will identify drivers of response and resistance.

De-identified biospecimens from 10 patients will be shipped to Personalis, Inc (Menlo Park, CA) for a purchased commercial service. Personalis, Inc. will only perform a fee-for-service. They will not retain any rights to data or biospecimens, they will not be otherwise engaged or involved as collaborators, and they will return any unused biospecimens upon completion of the service.

Results from the sequencing studies will not be released to the patients. These studies are for research purposes only and are not using a clinically validated platform.

9. STUDY CALENDAR

Following neoadjuvant therapy with cabozantinib and nivolumab, patients determined to be surgical candidates should be scheduled for surgery at least 28 days after their last dose of treatment with nivolumab or cabozantinib. For patients determined to be candidates for surgery, the surgical resection should be performed within 60 days of surgical evaluation.

	Pre-study	Lead In	Dose 1	Dose 2	Dose 3	Dose 4	Surgical Evaluation [15]	EOT Visit [15]
Study day	-28 to -1	0-13	14	28	42	56	70	30 days after last treatment dose
Visit Window[1]			+/-3	+/-3	+/-3	+/-3	+/-14	+/- 14
Nivolumab			X	X	X	X		
Cabozantinib		[——————————Once daily——————————]						
Surgical evaluation	X						X [15]	
Informed consent	X							
Inclusion/exclusion criteria	X							
Demographics	X							
Medical History[2]	X							
Concurrent medications	X		X	X	X	X		X
Vital Signs and pulse ox[3]	X		X	X	X	X		X
Physical exam[4]	X		X	X	X	X		X
Performance status	X		X	X	X	X		X
Height	X							
Weight	X		X	X	X	X		X
CBC with differential[5]	X		X	X	X	X		X
CMP[5,6]	X		X	X	X	X		X
TSH[5,7]	X		X	X	X	X		X
EKG	X		X	X	X	X		
Urinalysis and UPCR	X		X	X	X	X		X
Serum/Urine Preg[9]	X		X	X	X	X		
Urinalysis and microscopic exam	X		X	X	X	X		X
AFP[5]	X		X	X	X	X		X
LDH	X		X	X	X	X		X
INR and pTT	X		X	X	X	X		X
Lipase	X							
Hepatitis C[10]	X							
Hepatitis B[10]	X							
Viral quantitation[10]	X			X		X		X
Adverse event evaluation	X		X	X	X	X		X
Whole blood for research (Up to 150 CCs)[11]	X		X			X		
Medication diary (**Appendix C**)			X	X	X	X		
Tumor measurements[12]	X						X	
Tumor biopsy[11,13]	X		X					
Surgical specimen[14]								X

1) Longer delays to be approved by the principal investigator.
2) Includes history of lung disease, HIV, hepatitis B or C infection, and complete cancer history.
3) Temperature, respiration rate, blood pressure, pulse, and pulse oximetry should be taken prior to the administration of nivolumab.

4) Complete physical exam will be completed at baseline; focused physical examinations will be conducted thereafter. Exams, concomitant medication, AE assessments can be made up to 3 days prior to infusion.
5) Labs may be collected within a window of up to 7 days prior to initiating cabozantinib therapy, and before each dose of nivolumab therapy. Unexpected Grade 3 or greater laboratory abnormalities should be repeated within 24-72 hours if clinically indicated and monitored as necessary to determine if event meets toxicity criteria.
6) Albumin, alkaline phosphatase, total bilirubin, bicarbonate, BUN, calcium, chloride, creatinine, glucose, potassium, total protein, SGOT [AST], SGPT [ALT], sodium
7) Free T3/T4 to be checked reflexively if TSH is abnormal
8) EKG should be performed within 7 days prior to initiating cabozantinib
9) Serum or urine bHCG for women of childbearing potential only. Women with modestly elevated bHCG must obtain a negative transvaginal ultrasound in order to enroll on this protocol.
10) All screened patients should receive qualitative HBsAg, anti-HBc, anti-HBs, and anti-HCV. Patients found to have hepatitis B infection should receive quantitative hepatitis B Virus (HBV) DNA testing, and patients with hepatitis C infection should receive quantitative HCV RNA testing.
11) After eligibility is confirmed, but prior to first dose of therapy.
12) Tumor measurement will be performed with a liver protocol contrast CT, PET-CT, or or MRI (preferred). Patients will preferably have the same imaging modality used at baseline and end of treatment. Prior to starting treatment, patients should undergo imaging of the chest/abdomen/pelvis to exclude distal metastasis.
13) Only for subjects with tumor that is safely accessible for biopsy: A biopsy should be performed prior to initiation of cabozantinib therapy, and an on-treatment biopsy should be performed prior to nivolumab dose 1. Patients with tumor that is unsafe to biopsy do not require a biopsy for this protocol. To minimize risks of bleeding while the patient is receiving cabozantinib, the on-treatment biopsy should be performed with a 20 gauge (or smaller) needle.
14) Only for subjects who undergo surgical resection: Tumor tissue from the surgical resection will be collected, at the time of surgical resection. Refer to laboratory manual for details.
15) Surgical evaluation is standard of care, and is at the discretion of the treating surgical oncologist. Window provided for surgical evaluation is a recommendation only and is not mandated per protocol. EOT visit should occur prior to surgical resection when possible. Subjects who discontinue from treatment should be contacted every three months (+/- 2 weeks) to monitor overall survival. Information of other cancer therapies after discontinuation from the study treatment will be collected as well. Subjects who discontinue treatment should be contacted by telephone or email at 100 days (+ 14 day reporting window) to assess for treatment related toxicities that occur in the follow-up period.

10. MEASUREMENT OF EFFECT

10.1 Definitions

10.1.1 Evaluable

Evaluable for toxicity. All patients will be evaluable for toxicity from the time of their first treatment with cabozantinib or nivolumab.

Evaluable for response. All subjects who have received at least one dose of cabozantinib or nivolumab as part of this study and undergo a surgical evaluation are evaluable for response. Methods for evaluation of response are described below.

10.1.2 R0 resection rate

R0 resection is defined by a microscopically margin-negative resection, in which no tumor (gross or microscopic) remains in the primary tumor bed.

10.1.3 pCR rate in surgical specimens

The pCR rate is defined as the proportion of patients who receive a definitive surgical resection with no residual cancer in the primary pancreatic tissue or nodes (ypT0ypN0).

10.1.4 Disease Free Survival (DFS)

DFS is defined as the duration of time from start of enrollment in study to identification of recurrent disease on imaging or death, whichever occurs first. The pattern of recurrence (local vs. metastatic, or both) will also be inquired. Individuals will be censored at the date of the last scan if no event occurs.

10.1.5 Overall Survival (OS)

OS is defined as the duration of time from start of enrollment in study to time of death. Individuals will be censored at the date of the last contact if no event occurs.

10.1.6 Overall Response Rate (ORR)

ORR is defined as the proportion of patients achieving a complete response (CR) or partial response (PR) based on the revised Response Evaluation Criteria in Solid Tumors (RECIST) guideline (version 1.1) and by immune-related response criteria (irRC). Please refer to **Appendix F and G** for additional information. Changes in the largest diameter (unidimensional measurement) of the tumor lesions and the shortest diameter in the case of malignant lymph nodes are used in the RECIST 1.1 criteria.

All measurements should be taken and recorded in metric notation using a ruler or calipers. All baseline evaluations should be performed as closely as possible to the time of study enrollment. The same method of assessment and the same technique should be used to characterize each identified and reported lesion at the time of study enrollment and during follow-up. Imaging-based evaluation is preferred to evaluation by clinical examination unless the lesion(s) being followed cannot be imaged but are assessable by clinical exam.

Conventional CT and MRI: This guideline has defined measurability of lesions on CT scan based on the assumption that CT slice thickness is 5 mm or less. If CT scans have slice thickness greater than 5 mm, the minimum size for a measurable lesion should be twice the slice thickness. MRI has excellent contrast, spatial, and temporal resolution; however, there are many image acquisition variables involved in MRI, which greatly impact image quality, lesion conspicuity, and measurement. As with CT, if an MRI is performed, the technical

specifications of the scanning sequences used should be optimized for the evaluation of the type and site of disease. Furthermore, as with CT, the modality used at follow-up should be the same as was used at study enrollment and the lesions should be measured/assessed on the same pulse sequence.

11. DATA REPORTING / REGULATORY REQUIREMENTS

Adverse event guidelines and instructions for AE reporting can be found in **Section 7.0 (Adverse Events: List and Reporting Requirements)**.

11.1 Data Quality Assurance and Management

All information will be collected on study-specific case report forms (CRFs) by study staff. These data will be reviewed for completeness and accuracy by the Principal Investigator at each site.

11.2 Safety Meetings

Scheduled meetings will take place weekly and will include the protocol principal investigator, study coordinator(s), data manager(s), sub-investigators (as appropriate), collaborators (as appropriate), and biostatisticians (as appropriate) involved with the conduct of the protocol. During these meetings matters related to the following will be discussed: safety of protocol participants, validity and integrity of the data, enrollment rate relative to expectation, characteristics of participants, retention of participants, adherence to protocol (potential or real protocol violations), data completeness, and progress of data for objectives.

11.3 Monitoring

This is a DSMP Level II study under the Johns Hopkins Sidney Kimmel Comprehensive Cancer Center (SKCCC) Data Safety Monitoring Plan (DSMP, 12/6/2012). Eligibility will be monitored by the JHU SKCCC CRO. Data monitoring of this protocol will occur on a regular basis with the frequency dependent on the rate of subject accrual and the progress of the study. The protocol will be monitored internally by the Principal Investigator. The protocol will be monitored externally by the SKCCC CRO in accordance with SKCCC guidelines. Additional data and safety monitoring oversight will also be performed by the SKCCC Safety Monitoring Committee (SMC - as defined in the DSMP).

Dr. Laheru is holding the IND for this study. He will comply with all regulated reporting requirements to the FDA.

12. PRESENTATION OF STUDY DATA AND STUDY TERMINATION

12.1 Publication of Data and Protection of Trade Secrets

The Principal Investigator (Protocol Chair) holds the primary responsibility for publication of the study results; provided that the PI will provide any such publication to Exelixis, Inc. for review at least sixty (60) days before submission and also comply with any provisions

regarding publication that are agreed to between the PI's institution (eg, institution name.) and Exelixis, Inc. in the Clinical Trial Agreement related to this study. The results will be made public within 24 months of the end of data collection. However, if a report is planned to be published in a peer-reviewed journal, then that initial release may be an abstract that meets the requirements of the International Committee of Medical Journal Editors. In any event, a full report of the outcomes should be made public no later than three (3) years after the end of data collection. Authorship for abstracts and manuscripts resulting from this study will be determined in accordance to guidelines established by the International Committee of Medical Journal Editors.

12.2 Conditions for Terminating the Study

Exelixis reserves the right to terminate the study, and investigators reserve the right to terminate their participation in the study, at any time. Should this be necessary, Exelixis and the investigator will arrange the procedures on an individual study basis after review and consultation. In terminating the study, Exelixis and the investigator will ensure that adequate consideration is given to the protection of the subjects' interests.

13. STATISTICAL CONSIDERATIONS

This is a single arm trial to evaluate the safety and feasibility of neoadjuvant cabozantinib plus nivolumab (CaboNivo) for treating patients with locally advanced HCC.

13.1 Primary Endpoint

The primary endpoint focuses on feasibility, which will be measured by the proportion of patients that experience a treatment-related AE that precludes continuing on to surgery within 60 days of the planned date for surgical evaluation. This outcome specifically focuses on whether or not the treatment is a barrier to surgery. Individuals who do not go on to receive surgery due to other causes (e.g. progressive disease, patient refusal) will not be counted as failures since none of the patients would be expected to proceed to surgery under the standard of care. The proportion of failures will be calculated with an exact Binomial 95% confidence interval.

13.2 Safety Endpoints

The safety analysis will be performed in all treated patients. AE data will be listed individually and summarized by system organ class and preferred terms within a system organ class, grade (based upon the National Cancer Institute's CTCAE Version 4.03), relatedness to treatment, and expectedness. Additional details on the process for classifying AEs are provided in **Section 7.3**. Summary statistics will include counts and proportions as well as rates with 95% confidence intervals based upon a Negative Binomial distribution. Both person-level and event-level summaries will be included.

The proportion of patients with unacceptable toxicities (See **Section 5.6**) will be monitored continuously throughout the trial using a Bayesian stopping guideline. A Beta(1.5, 5.5) prior, which mirrors but is slightly more conservative than the threshold establishing the maximum tolerated dose (at most 1 toxicity out of 6 patients) in standard 3+3 designs. The prior represents a toxicity rate of 21% and an effective sample size of 7 patients (Morita *et al*, 2016). The prior

was chosen to represent a higher rate than the expect rate of 10% in order to be conservative. Assuming a binomial likelihood distribution for the observed trial data, the posterior distribution for the probability of an unacceptable toxicity, p, would follow a Beta(1.5+x, 5.5 + N – x) distribution, where x represents the number of unacceptable toxicities out of N patients. Monitoring will begin at N = 3 and continue for all patients thereafter. We would consider a 30% toxicity rate to be unacceptable. The criteria for re-evaluation at each sample size (N = 3-15) were selected to achieve a high probability of stopping at unacceptable toxicity levels with a low probability of stopping if the toxicity levels are in the expected 10% range. The therapy will be re-evaluated if the posterior probability that the toxicity rate exceeds 25% is greater than 50%. The number needed to trigger re-evaluation using this rule was calculated by finding the smallest number of toxicities such that the Pr(p > 0.25 | p ~ Beta(1.5 + x, 5.5+N-x)) > 0.5 was calculated for N = 3 to 15. **Table 6** summarizes the stopping boundaries starting with the initial cohort of 3 patients through the maximum sample size of 15 patients along with the posterior probabilities for each threshold.

Table 6. The number of unacceptable toxicities needed to trigger stopping guidelines throughout the course of the study.

Number of Patients	Number Needed To Trigger Re-evaluation	Posterior probability that toxicity rate exceeds 50%
3	2	0.73
4		0.66
5		0.59
6		0.52
7	3	0.70
8		0.64
9		0.58
10		0.52
11	4	0.68
12		0.62
13		0.57
14		0.52
15	5	0.66

The probability of triggering the stopping guidelines was assessed for a range of possible toxicity rates using simulations with 10000 replicates (**Table 7)**. The probability of stopping to re-evaluate was 14.6% if the true proportion with an unacceptable toxicity was 10%. In comparison, the probability of stopping early was 76% if the true proportion with an unacceptable toxicity was 30%.

Table 7. Probability of triggering a re-evaluation for a range of values for the true underlying toxicity rate.

True probability of unacceptable toxicity	Probability of triggering stopping guidelines
5%	3.8%
10%	14.6%
12%	20%
15%	30%
20%	47%
25%	63%
30%	76%

13.3 Sample Size/Accrual Rate

A total of 15 patients will be enrolled over the course of 12 months (i.e. 1-2 patients/month). The primary goal is to establish feasibility based upon the number of patients who do no experience a treatment-related AE that precludes continuing on to surgery. The primary endpoint will be evaluated in all patients that receive at least one dose of treatment.

A failure rate of more than 33% (>5/15) would be considered unacceptable, i.e. we would conclude that the treatment was not feasible if 5 or more of the first 15 met the criteria for failure (see table 6). The probability of concluding that the treatment is feasible was calculated using exact Binomial probabilities, i.e. $Pr(X > 5 \mid X \sim Binomial(15\ p))$, where X is the number of successes out of 15 and p is the underlying probability of success, for a range of potential underlying success rates (**Table 8**). We expect that approximately 2/15 patients (13%) will meet this criteria (i.e. 2 patients will have a failure). If this were the true rate, the probability of concluding that the study was feasible would be 96%. Conversely, if the true rate were 50%, the probability of concluding that the treatment was feasible would only be 6%.

Table 8. Probability of saying the treatment is feasible, i.e. observing 4 or more failures out of 15, for a range of possible values for the true underlying probability of failure.

Underlying Probability of Failure (p)	Probability Conclude Feasible
60%	< 1%
50%	6%
40%	22%
30%	52%
20%	84%
13%	96%

Under the standard of care, no patients would be expected to go on to receive surgery. In addition to monitoring the proportion of patients with treatment-related AEs that preclude toxicity, we will also monitor the number of individuals who go on to receive surgery as part of our determination of feasibility. If none of the patients (0/15) go on to receive surgery, then we would have evidence that the proportion that could receive surgery was significantly less than 18.2%, the upper boundary of the exact binomial 95% confidence interval, and the treatment would not be considered feasible.

13.4 Stratification Factors

The study has a single treatment arm. There are no stratification factors.

13.5 Analysis of Secondary and Exploratory Endpoints

A complete list of secondary and exploratory endpoints is provided in **Section 2.5** and **Section 2.6**, respectively. Binary outcomes (e.g. percent who receive an R0 surgical resection, response) will be summarized using counts, percentages and exact Binomial confidence intervals. Logistic regression will be used to explore the potential impact of clinical and demographic risk factors; although the power for these investigations is limited due to the small sample size.

The two primary time-to-event outcomes are overall survival and disease free survival. Overall survival is defined as the time from study initiation to death. Individuals will be censored at the date of last contact if death has not occurred at the time of analysis. Disease free survival (DFS) will be evaluated in the subset that received surgery. DFS is defined as the time from surgery to recurrence or death. Individuals will be censored at the date of the last evaluation of recurrence if no event has occurred at the time of analysis. Time-to-event outcomes will be analyzed using Kaplan-Meier estimates of the survival function, log-rank tests and Cox proportional hazards models. The later techniques will be exploratory in nature and will have limited power due to the small sample size.

The correlative studies will focus on the paired pre-treatment and pre-nivolumab biopsies. Plots will be used to summarize changes for each individual and for the population as a whole. Comparisons between the time points will be made using paired t-tests (or Wilcoxon rank-sum tests if appropriate) for continuous variables and McNemar's test for binary or categorical variables. In addition to biopsies, serial laboratory measurements will be taken throughout the course of follow-up. The graphical tools and comparisons of two specific time-points will use the techniques described for the biopsies. In addition, longitudinal modeling techniques (e.g. GEE and linear mixed effects models) that take into account the correlation between repeated measurements will be explored.

Genomic sequencing library construction, whole genome/exome sequencing, whole transcriptome sequencing, microbial sequencing, neoepitope prediction, mutation burden, and bioinformatic analysis will be performed either at an on-campus laboratory or at an off-campus sequencing service. All the samples will be de-identified before sending to any laboratory for sequencing. The FASTQ files, BAM files and VCF files will be generated and analyzed. Genomic sequencing data will be stored and computations conducted using a JH IT managed subscription of Azure.

13.6 Reporting and Exclusions

13.6.1 Evaluation of Toxicity

All patients will be evaluable for toxicity from the time of their first treatment with cabozantinib or nivolumab.

13.6.2 Evaluation of Response

All patients included in the study must be assessed for response to treatment, even if there are major protocol treatment deviations or if they are ineligible. Each patient will be assigned one of the following categories: 1) complete response, 2) partial response, 3) stable disease, 4) progressive disease, 5) early death from malignant disease, 6) early death from toxicity, 7) early death because of other cause, or 9) unknown (not assessable, insufficient data). [Note: By arbitrary convention, category 9 usually designates the "unknown" status of any type of data in a clinical database.]

All of the patients who met the eligibility criteria (with the possible exception of those who received no study medication) should be included in the main analysis of the response rate. Patients in response categories 4-9 should be considered to have a treatment failure (disease progression). Thus, an incorrect treatment schedule or drug administration does not result in exclusion from the analysis of the response rate. Precise definitions for categories 4-9 will be protocol specific.

All conclusions should be based on all eligible patients. Sub-analyses may then be performed on the basis of a subset of patients, excluding those for whom major protocol deviations have been identified (*e.g.*, early death due to other reasons, early discontinuation of treatment, major protocol violations, etc.). However, these subanalyses may not serve as the basis for drawing conclusions concerning treatment efficacy, and the reasons for excluding patients from the analysis should be clearly reported. The 95% confidence intervals should also be provided.

REFERENCES

Apolo AB, Mortazavi A, Stein M, Pal SK, Davarpanah N, Parnes HL, Ning YM, Francis DC, Cordes LM, Berniger M, Steinberg SM, Monk P, Lancaster T, Mayer T, Costello R, Bottaro DP, Dahut WL (2016) A phase I study of cabozantinib plus nivolumab (CaboNivo) in patients (pts) refractory metastatic urothelial carcinoma (mUC) and other genitourinary (GU) tumors. *Ann Oncol* **27**: 774PD, doi:10.1093/ANNONC/MDW373.02.

Belghiti J, Panis Y, Farges O, Benhamou JP, Fekete F (1991) Intrahepatic recurrence after resection of hepatocellular carcinoma complicating cirrhosis. *Ann Surg* **214**: 114–117.

Bouattour M, Fartoux L, Rosmorduc O, Scatton O, Vibert E, Costentin C, Soubrane O, Ronot M, Granier MM, De Gramont A, Belghiti J, Paradis V, Wendum D, Tijeras-Raballand A, Hadengue A, Brusquant D, Chibaudel B, Raymond E, Faivre SJ (2016) BIOSHARE multicenter neoadjuvant phase 2 study: Results of pre-operative sorafenib in patients with resectable hepatocellular carcinoma (HCC). *ASCO Annu Meet Proc* **34**:.

Chen MF, Hwang TL, Jeng LB, Wang CS, Jan YY, Chen SC (1994) Postoperative recurrence of hepatocellular carcinoma. Two hundred five consecutive patients who underwent hepatic resection in 15 years. *Arch Surg* **129**: 738–742.

Gabrilovich DI, Nagaraj S (2009) Myeloid-derived suppressor cells as regulators of the immune system. *Nat Rev Immunol* **9**: 162–174, doi:10.1038/nri2506.

Goyal L, Muzumdar MD, Zhu AX (2013) Targeting the HGF/c-MET Pathway in Hepatocellular Carcinoma. *Clin Cancer Res* **19**: 2310–2318, doi:10.1158/1078-0432.CCR-12-2791.

Lise M, Bacchetti S, Da Pian P, Nitti D, Pilati PL, Pigato P (1998) Prognostic factors affecting long term outcome after liver resection for hepatocellular carcinoma: results in a series of 100 Italian patients. *Cancer* **82**: 1028–1036.

Morita S, Thall PF, Müller P (2008) Determining the effective sample size of a parametric prior. Biometrics. **64**: 595-602.

Park J-W, Chen M, Colombo M, Roberts LR, Schwartz M, Chen P-J, Kudo M, Johnson P, Wagner S, Orsini LS, Sherman M (2015) Global patterns of hepatocellular carcinoma management from diagnosis to death: the BRIDGE Study. *Liver Int* **35**: 2155–2166, doi:10.1111/liv.12818.

Portolani N, Coniglio A, Ghidoni S, Giovanelli M, Benetti A, Tiberio GAM, Giulini SM (2006) Early and late recurrence after liver resection for hepatocellular carcinoma: prognostic and therapeutic implications. *Ann Surg* **243**: 229–235, doi:10.1097/01.sla.0000197706.21803.a1.

Tabrizian P, Jibara G, Shrager B, Schwartz M, Roayaie S (2015) Recurrence of Hepatocellular Cancer After Resection. *Ann Surg* **261**: 947–955, doi:10.1097/SLA.0000000000000710.

Yen BL, Yen M-L, Hsu P-J, Liu K-J, Wang C-J, Bai C-H, Sytwu H-K (2013) Multipotent Human Mesenchymal Stromal Cells Mediate Expansion of Myeloid-Derived Suppressor Cells via Hepatocyte Growth Factor/c-Met and STAT3. *Stem Cell Reports* **1**: 139–151,

doi:10.1016/j.stemcr.2013.06.006.

Zhou XD, Tang ZY, Yang BH, Lin ZY, Ma ZC, Ye SL, Wu ZQ, Fan J, Qin LX, Zheng BH (2001) Experience of 1000 patients who underwent hepatectomy for small hepatocellular carcinoma. *Cancer* **91**: 1479–1486.

APPENDIX A PERFORMANCE STATUS CRITERIA

ECOG Performance Status Scale		Karnofsky Performance Scale	
Grade	Descriptions	Percent	Description
0	Normal activity. Fully active, able to carry on all pre-disease performance without restriction.	100	Normal, no complaints, no evidence of disease.
		90	Able to carry on normal activity; minor signs or symptoms of disease.
1	Symptoms, but ambulatory. Restricted in physically strenuous activity, but ambulatory and able to carry out work of a light or sedentary nature (*e.g.*, light housework, office work).	80	Normal activity with effort; some signs or symptoms of disease.
		70	Cares for self, unable to carry on normal activity or to do active work.
2	In bed <50% of the time. Ambulatory and capable of all self-care, but unable to carry out any work activities. Up and about more than 50% of waking hours.	60	Requires occasional assistance, but is able to care for most of his/her needs.
		50	Requires considerable assistance and frequent medical care.
3	In bed >50% of the time. Capable of only limited self-care, confined to bed or chair more than 50% of waking hours.	40	Disabled, requires special care and assistance.
		30	Severely disabled, hospitalization indicated. Death not imminent.
4	100% bedridden. Completely disabled. Cannot carry on any self-care. Totally confined to bed or chair.	20	Very sick, hospitalization indicated. Death not imminent.
		10	Moribund, fatal processes progressing rapidly.
5	Dead.	0	Dead.

APPENDIX B PATIENT DRUG INFORMATION HANDOUT AND WALLET CARD

Information for Patients, Their Caregivers, and Non-Study Healthcare Team on Possible Interactions with Other Drugs and Herbal Supplements

The patient _____________________________ is enrolled on a clinical trial using the experimental study drugs cabozantinib and nivolumab. This form includes important information for others who care for this patient.

These are the things that you as a healthcare provider need to know:

Cabozantinib

Cabozantinib interacts with Cytochrome P450 3A4 (abbreviated CYP3A4) system and can also cause QTc prolongation. Cabozantinib may also increase a patient's risk of bleeding.

- Drugs or supplements that either inhibit or induce CYP3A4 may affect the level of cabozantinib and should be avoided. Some drugs that should specifically be avoided include:
  - Strong inhibitors of CYP3A4 (eg, ketoconazole, itraconazole, clarithromycin, indinavir, nefazodone, nelfinavir, and ritonavir)
  - Strong inducers of the CYP3A4 (eg, phenytoin, carbamazepine, rifampin, rifabutin, rifapentine, phenobarbital, and St. John's Wort) may significantly decrease cabozantinib concentrations and should be avoided.
  - Grapefruit may also increase plasma concentrations of cabozantinib and should be avoided.
- The study doctor may be concerned about QTc prolongation, and use of medicines that are associated with greater risk for having QTc prolongation should be avoided.
- Cabozantinib should be used with caution in combination with blood thinners.

Nivolumab

Nivolumab is a type of immune therapy. There is a risk of developing autoimmune side effects from nivolumab, and these autoimmune side effects may be serious and challenging to diagnose. Examples of autoimmune side effects previously observed in clinical trials of immunotherapy drugs include:

- Endocrinopathies (eg, hypothyroidism, adrenal insufficiency, panhypopituitarism, new onset type 1 diabetes)
- Inflammation of various organs (colitis, pneumonitis, hepatitis, myocarditis)
- Rheumatologic diseases (for example, rheumatoid arthritis, myositis)

When evaluating this patient for an acute medical complaint, please consider that the patient may be experiencing an immune-related adverse event.

To the patient: Take this paper with you to your medical appointments and keep the attached information card in your wallet.

Cabozantinib and nivolumab may interact with other drugs, which can cause side effects. For this reason, it is very important to tell your study doctors of any medicines you are taking before you enroll onto this clinical trial. It is also very important to tell your doctors if you stop taking any regular medicines, or if you start taking a new medicine while you take part in this study. When you talk about your current medications with your doctors, include medicine you buy without a prescription (over-the-counter remedy), or any herbal supplements such as St. John's Wort. It is helpful to bring your medication bottles or an updated medication list with you.

These are the things that you and they need to know:

- Please be very careful! Over-the-counter drugs (including herbal supplements) may contain ingredients that could interact with your study drug. Speak to your doctors or pharmacist to determine if there could be any side effects.
- Your regular health care provider should check a frequently updated medical reference or call your study doctor before prescribing any new medicine or discontinuing any medicine. Your study doctor's name is

______________________________________ and he or she can be contacted at

______________________________________.

**STUDY DRUG INFORMATION WALLET CARD**<br>You are enrolled on a clinical trial using the experimental study drugs nivolumab and cabozantinib. Cabozantinib may interact with drugs that are processed by your liver, and can also affect the electrical activity of your heart. Because of this, it is very important to:<br>1) Tell your doctors if you stop taking any medicines or if you start taking any new medicines.<br><br>2) Tell all of your health care providers (doctors, physician assistants, nurse practitioners, or pharmacists) that you are taking part in a clinical trial.<br><br>3) Check with your doctor or pharmacist whenever you need to use an over-the-counter medicine or herbal supplement.	▪ Cabozantinib interacts with a specific liver enzyme called CYP3A4, can affect the heart's electrical activity (QTc prolongation], and can increase bleeding risk. It must be used very carefully with other medicines that interact with CYP3A4, that cause QTc prolongation, or with blood thinners.<br>▪ Nivolumab is a type of immunotherapy can cause immune-related adverse events. If you are evaluated for an urgent medical problem, tell your treatment team that you could be experiencing an immune-related adverse event.<br>▪ Before prescribing new medicines, your regular health care providers should go to a frequently updated medical reference for a list of drugs to avoid, or contact your study doctor.<br><br>Your study doctor's name is ______________________________<br><br>and can be contacted at _________________________________.

APPENDIX C PATIENT MEDICATION DIARY

INSTRUCTIONS TO THE PATIENT:
1. Complete one form for each 2 week-period while you take **cabozantinib**.
2. Take your dose of **cabozantinib** at about the same time each morning on an empty stomach You cannot eat for at least 2 hours before or 1 hour after taking cabozantinib. The pills should be swallowed whole and must not be crushed or broken.
3. Record the date, the number of pills you took, and when you took them. Record doses as soon as you take them; do not batch entries together at a later time.
4. If you miss taking your dose and remember the dose can be taken within 12 hours of when it should have been taken. Resume dosing with the next scheduled dose.
5. If you have any comments or notice any side effects, please record them in the Comments column. If you make a mistake while you write, please cross it out with one line, put your initials next to it, and then write the corrected information next to your initials. Example: ~~10:30 am~~ *SB 9:30 am*
6. Please return this form to your physician when you go for your next appointment.

Day	Date	Time of Daily Dose	# of Tablets Taken	Comments
0				
1				
2				
3				
4				
5				
6				
7				
8				
9				
10				
11				
12				
13				
14				
15				
16				
17				
18				
19				
20				
21				
22				

23				
24				
25				
26				
27				
28				
Participant's Signature			Date	

Physician's Office will complete this section:

1. Total number of tablets taken this month (each size) ____

2. Physician/Nurse/Data Manager's Signature/Date ____

INSTRUCTIONS TO THE PATIENT:

1. Complete one form for each 2 week-period while you take **cabozantinib**.
2. Take your dose of **cabozantinib** at about the same time each morning on an empty stomach You cannot eat for at least 2 hours before or 1 hour after taking cabozantinib. The pills should be swallowed whole and must not be crushed or broken.
3. Record the date, the number of pills you took, and when you took them. Record doses as soon as you take them; do not batch entries together at a later time.
4. If you miss taking your dose and remember the dose can be taken within 12 hours of when it should have been taken. Resume dosing with the next scheduled dose.
5. If you have any comments or notice any side effects, please record them in the Comments column. If you make a mistake while you write, please cross it out with one line, put your initials next to it, and then write the corrected information next to your initials. Example: ~~10:30 am~~ *SB 9:30 am*
6. Please return this form to your physician when you go for your next appointment.

Day	Date	Time of Daily Dose	# of Tablets Taken	Comments
29				
30				
31				
32				
33				
34				
35				
36				
37				
38				
39				
40				
41				
42				
43				
44				
45				
46				
47				
48				
49				
50				
51				
52				
53				

54				
55				
56				
Participant's Signature			Date	

Physician's Office will complete this section:

1. Total number of tablets taken this month (each size)
____

2. Physician/Nurse/Data Manager's Signature/Date
____

APPENDIX D RECOMMENDED MANAGEMENT ALGORITHMS

These general guidelines constitute guidance to the Investigator and may be supplemented by discussions with the Protocol Chair representing the IND Sponsor. The guidance applies to all immuno-oncology (I-O) agents and regimens.

A general principle is that differential diagnoses should be diligently evaluated according to standard medical practice. Non-inflammatory etiologies should be considered and appropriately treated.

Corticosteroids are a primary therapy for immuno-oncology drug-related adverse events. The oral equivalent of the recommended IV doses may be considered for ambulatory subjects with low-grade toxicity. The lower bioavailability of oral corticosteroids should be taken into account when switching to the equivalent dose of oral corticosteroids.

Consultation with a medical or surgical specialist, especially prior to an invasive diagnostic or therapeutic procedure, is recommended.

The frequency and severity of the related adverse events covered by these algorithms will depend on the immuno-oncology agent or regimen being used.

Dermatitis/Skin Rash

All subjects on study should be advised on prophylactic skin care. This includes the use of hypoallergenic moisturizing creams, ointment for dry skin, and sunscreen with sun protection factor ≥ 30. A dermatologist should evaluate any persistent and/or severe rash or pruritus. A skin biopsy should be performed, unless contraindicated.

Grade 1	– Continue nivolumab and cabozantinib.<br>– Initiate symptomatic therapy with antihistamine PRN.<br>– Consider topical steroids and/or other symptomatic therapy (e.g., antihistamines).
Grade 2	– Continue nivolumab and cabozantinib.<br>– Consider consultation with a dermatologist.<br>– Administer topical steroids.<br>– Consider higher potency topical steroids if rash does not improve.
Grade 3	– Hold nivolumab and cabozantinib.<br>– Consult a dermatologist.<br>– Administer 1.0-2.0 mg/kg/day IV methylprednisolone IV or IV equivalent if the rash is at least possibly attributable to the nivolumab therapy.<br>– Restart nivolumab at fixed dose and cabozantinib at 1 dose reduction if rash resolves to ≤ tolerable grade 2, and systemic steroid dose is < 10 mg oral prednisone equivalent per day.
Grade 4	– Permanently discontinue nivolumab and cabozantinib. Patient may not resume treatment, regardless of benefit.<br>Consult a dermatologist.<br>– Administer 1.0-2.0 mg/kg/day IV methylprednisolone IV or IV equivalent. Taper steroids over at least 1 month and add prophylatic antibiootics for opportunistic infections.

Diarrhea
Subjects should be instructed to notify their physician immediately at the first signs of poorly formed or loose stool or an increased frequency of bowel movements. Administration of antidiarrheal/antimotility agents is recommended at the first sign of diarrhea as initial management. Some subjects may require concomitant treatment with more than one antidiarrheal agent. A suggested regimen is:

Loperamide: Initiate dose with 4 mg, then 4 mg/6 hr around the clock, alternating with Lomotil.
Lomotil (diphenoxylate and atropine): 2 tablets (diphenoxylate 5 mg) every 6 hr around the clock

In addition, general supportive measures should be implemented including hydration, correction of fluid and electrolyte abnormalities, small frequent meals, and stopping lactose-containing products, high fat meals, and alcohol. For events of significant duration or magnitude or associated with signs of systemic inflammation or acute-phase reactants (e.g., increased CRP, platelet count, or bandemia): Perform sigmoidoscopy (or colonoscopy, if appropriate) with colonic biopsy, with three to five specimens for standard paraffin block to check for inflammation and lymphocytic infiltrates to confirm colitis diagnosis.

Grade 1	– Continue nivolumab and cabozantinib. Initiate symptomatic treatment.<br>– Endoscopy is recommended if symptoms persist for >7 days.
Grade 2	– Hold nivolumab and cabozantinib, and initiate symptomatic treatment.<br>– If Grade 2 diarrhea persists after 48 hr total treatment with Lomotil and loperamide, consider second-line agents (e.g., octreotide, budesonide, tincture of opium).<br>– Patient referral to GI specialist is recommended.<br>– For events that persist >7 days, initiate treatment with 1–2 mg/kg/day oral prednisone or equivalent. If worsens or persists >5 days with steroids, treat as grade 3-4.<br>– Resumption of nivolumab and cabozantinib at one dose reduction be considered, after consultation with the trial PI, in patients who have fully recovered.
Grade 3-4	– Permanently discontinue nivolumab and cabozantinib.<br>– Refer patient to GI specialist for evaluation and consider lower endoscopy and biopsy.<br>– Initiate treatment with 1–2 mg/kg/day intravenous methylprednisolone or IV equivalent. If symptoms persist >3-5 days, or recur after improvement, add infliximab 5 mg/kg. NOTE: infliximab should not be used in cases of perforation or sepsis.<br>– If symptoms improve, continue steroids until grade 1, then taper over at least 1 month.

Endocrine Disorders
Changes in thyroid function tests (TFTs) and hypothyroidism have been reported with cabozantinib as well as nivolumab.

Grade 1 (asymptomatic)	– Continue nivolumab and cabozantinib.

thyroid disorder	– Follow local guidelines for initiating treatment with thyroid replacement medication, if appropriate.
Grade 2+ (symptomatic) thyroid disorder	– Hold nivolumab and cabozantinib.<br>– Initiate treatment with anti-thyroid drug such as methimazole or carbimazole for symptomatic hyperthyroidism, or start thyroid-replacement hormone for symptomatic hypothyroidism.<br>– Consider patient referral to endocrinologist.<br>– Resume nivolumab and cabozantinib when symptoms are controlled and thyroid function is improving. Permanently discontinue nivolumab for life-threatening immune-related hyperthyroidism.
Symptomatic endocrinopathy	– Evaluate endocrine function<br>– Consider pituitary scan<br><br><u>Symptomatic with abnormal lab/pituitary scan:</u><br>– Delay nivolumab. Initiate appropriate hormone therapy and 1-2 mg/kg/day methylprednisolone IV or PO equivalent<br><br><u>No abnormal lab/pituitary MRI scan but symptoms persist</u><br>– Repeat labs in 1-3 weeks and MRI in 1 month
Suspicion of adrenal crisis (severe dehydration, hypotension, shock out of proportion to current illness)	– Delay or discontinue treatment per protocol<br>– Stress dose IV steroids with mineralocorticoid activity<br>– Rule out sepsis and administer IV fluids<br>– Consult an endocrinologist<br>– If adrenal crisis is ruled out, then treat as above for symptomatic endocrinopathy

<u>Fatigue, anorexia, and weight loss</u>

Fatigue has been reported during treatment with cabozantinib as well as nivolumab. Common causes of fatigue such as anemia, deconditioning, emotional distress (depression and/or anxiety), poor nutrition, sleep disturbance, and hypothyroidism should be ruled out and/or these causes treated in accordance to standard of care. Individual non-pharmacological and/or pharmacologic interventions directed to the contributing and treatable factors should be given. Note: Chronic use of modafinil should be avoided because of its potential to reduce cabozantinib exposure (see Investigator's Brochure). Anorexia and weight loss should be managed in accordance to local standard of care including nutritional support. If fatigue, anorexia, or weight loss cannot be adequately managed, study treatment should be temporarily interrupted or dose of cabozantinib reduced.

<u>GI Perforation/Fistula and Non-GI Fistula Formation</u>

Gastrointestinal perforation/GI fistula: Prior to initiation of treatment with cabozantinib, subjects should be carefully evaluated for potential risk factors including (but not limited to) the following:

- Tumors invading GI or respiratory tracts
- Active peptic ulcer disease, inflammatory bowel disease (eg, ulcerative colitis, Crohn's disease), diverticulitis, cholecystitis or symptomatic cholangitis, or appendicitis
- History of abdominal fistula, GI perforation, bowel obstruction, or intra-abdominal abscess
- Ongoing visceral complications from prior radiation therapy

- Prior GI surgery (particularly when associated with delayed or incomplete healing)

Complete healing following abdominal surgery and/or resolution of intra-abdominal abscess must be confirmed prior to initiating treatment with cabozantinib.
After starting cabozantinib, subjects should be monitored for early signs of GI perforation such as abdominal pain, nausea, emesis, constipation, and fever especially if known risk factors for developing GI perforation or fistula are present.
Discontinue cabozantinib treatment in subjects who have been diagnosed with GI perforation/fistula.
Non-GI fistula formation: Complications from radiation therapy has been identified as a possible predisposing risk factor for fistula formation in subjects undergoing treatment with cabozantinib.
Subjects with any clinically relevant ongoing complications from prior radiation therapy (ie, radiation esophagitis or other inflammation of the viscera) should not be treated with cabozantinib.
Radiation therapy to the thoracic cavity (including mediastinum) should be avoided within 3 months of starting treatment with cabozantinib (excluding local radiation for bone metastases).
Fistula should be ruled out as appropriate in cases of onset of severe mucositis or difficulty swallowing after start of therapy. Discontinue cabozantinib and initiate appropriate management in subjects who have been diagnosed with a non-GI fistula.
Discontinue cabozantinib treatment in subjects who have been diagnosed with non-GI fistula.

Hematological Disorders
Hematological toxicities (ie, neutropenia and thrombocytopenia) and associated complications have been observed after administration of cabozantinib and may be managed with dose interruptions and/or dose reductions. Use of granulocyte colony-stimulating factor support for neutrophil recovery is allowed per investigator discretion and in accordance with accepted guidelines after the first incidence of clinically relevant cytopenia.
Complete blood counts with differentials and platelets should be performed regularly. Subjects with hematologic toxicities may require additional or more frequent laboratory tests according to institutional guidelines. Results of such tests are to be forwarded to the local laboratory data management vendor.
Febrile neutropenia or evidence of infection associated with neutropenia must be assessed immediately and treated appropriately and in a timely manner according to institutional guidelines.
Dose reductions or dose interruptions for anemia are not mandated but can be applied as clinically indicated. Supportive care such as red blood cell transfusions may be managed according to institutional guidelines.

Hemorrhagic Events
Hemorrhagic events have been reported with cabozantinib. In order to mitigate risk of severe hemorrhage, subjects should be evaluated for potential bleeding risk factors before initiating cabozantinib treatment and monitored for bleeding events with serial complete blood counts and physical examination while on study. Risk factors for hemorrhagic events may include (but may not be limited to) the following:

- Tumor of the lung with cavitary lesions or tumor lesions which invades, encases, major blood vessels. The anatomic location and characteristics of tumor as well as the medical history must be carefully reviewed in the selection of subjects for treatment with cabozantinib;
- Recent or concurrent radiation to the thoracic cavity;

- Active peptic ulcer disease, inflammatory GI diseases including Crohn's disease and ulcerative colitis;
- Underlying medical conditions which affect normal hemostasis (eg, deficiencies in clotting factors and/or platelet function, or thrombocytopenia);
- Concomitant medication with anticoagulants or other drugs which affect normal hemostasis;
- History of clinically significant hemoptysis.

The risk of hemorrhage in cabozantinib-treated subjects with brain metastases has not been thoroughly analyzed. Though the incidence of CNS hemorrhage events in a study of subjects with GB was higher than observed in general population of subjects with cancer treated with cabozantinib, it is not clear how the risk of hemorrhage in GB translates to a risk of hemorrhage for subjects with brain metastases. Currently, brain metastases of carcinomas are not contraindications to the use of cabozantinib, but subjects with brain metastases should be monitored with a high index of suspicion if symptoms that could be due to a CNS hemorrhage occur.
Cabozantinib should be discontinued in subjects with serious and life-threatening bleeding events or recent hemoptysis (≥ 2.5 mL of red blood).

Hepatotoxicity
Elevations of ALT, AST, and bilirubin have been observed during treatment with cabozantinib and nivolumab. It is recommended that subjects with elevation of ALT, AST, and/or bilirubin, or with right upper-quadrant abdominal pain and/or unexplained nausea or vomiting, have more frequent laboratory monitoring of live function tests. Concurrent medication, viral hepatitis, and toxic or neoplastic etiologies should be considered and addressed, as appropriate. If possible, hepatotoxic concomitant medications should be discontinued in subjects who develop increased values of ALT, AST, or bilirubin.

Grade 1	– Continue nivolumab and cabozantinib.<br>– Monitor LFTs more frequently until values resolve to baseline values.
Grade 2	– Hold cabozantinib and nivolumab. Monitor LFTs more frequently until values resolve to baseline values. If LFTs remain elevated for >7 days or worsen, consider treatment with 0.5-1 mg/kg/day methylprednisolone or oral equivalent and when LFTs return to grade 1 or baseline, taper steroids over at least 1 month.<br>– Consider restarting cabozantinib at 1 dose reduction after ALT, AST, and bilirubin levels resolve to at least Grade ≤ 1 or baseline.
Grade 3	– Hold nivolumab and cabozantinib.<br>Consider patient referral to GI specialist for evaluation and liver biopsy to establish etiology of hepatic injury.<br>Initiate treatment with 0.5-1 mg/kg/day methylprednisolone IV or PO equivalent. If event does not improve within 48 hours after initiating corticosteroids, consider adding an immunosuppressive agent (mycophenolate mofetil 1g BID).<br>– Permanently discontinue nivolumab and cabozantinib if does not improve to ≤ grade 1 severity within 1 week of starting therapy.<br>– In most cases, treatment will be permanently discontinued. Resumption of nivolumab and cabozantinib at one dose reduction may only be considered,

	after consultation with the trial PI, in patients who are deriving benefit, and only after ALT, AST, and bilirubin levels resolve to at least Grade ≤ 1 or baseline.
Grade 4	– Permanently discontinue nivolumab and cabozantinib.<br>– Consider patient referral to GI specialist for evaluation and liver biopsy to establish etiology of hepatic injury.<br>– Initiate treatment with 1–2 mg/kg/day methylprednisolone IV or IV equivalent.<br>If event does not improve within 48 hours after initiating corticosteroids, consider adding an immunosuppressive agent (mycophenolate mofetil 1g BID).<br>If event resolves to grade 1 or better, taper corticosteroids over ≥1 month.

Hypertension

Hypertension has been reported among subjects treated with cabozantinib. Treatment guidelines for hypertension deemed related to cabozantinib are presented in below. Blood pressure should be monitored in a constant position at each visit (either sitting or supine). In general, subjects with known hypertension should be optimally managed before study entry. Decisions to decrease or hold the dose of study treatment must be based on blood pressure readings taken by a medical professional and must be confirmed with a second measurement at least 5 minutes after the first measurement. Other than for hypertension requiring immediate therapy, the presence of new or worsened hypertension should be confirmed at a second visit before taking therapeutic action. It is recommended that this second visit occurs within 1 week. Cabozantinib should be discontinued in subjects with hypertensive crises or hypertensive encephalopathy.

Subjects NOT receiving optimized anti-hypertensive therapy	
> 150 mm Hg (systolic)[A] and < 160 mm Hg<br>OR<br>> 100 mm Hg (diastolic) and < 110 mm Hg	– Optimize antihypertensive medications by adding new or additional antihypertensive medications and/or increase dose of existing medications.<br>– Reduce cabozantinib by one dose level if optimal antihypertensive therapy (usually to include 3 agents) does not result in BP <150 mm Hg systolic or <100 mm Hg diastolic<br>– If subject is symptomatic interrupt study treatment
≥ 160 mm Hg (systolic)<br>OR<br>≥ 110 mm Hg (diastolic)	– Reduce cabozantinib by one dose level or interrupt study treatment per investigator discretion<br>– Add new or additional anti-hypertensive medications and/or increase dose of existing medications and monitor subject closely for hypotension. If optimized antihypertensive therapy (usually to include 3 agents) does not result in BP < 150 mm Hg systolic or < 100 mm Hg diastolic, study treatment should be dose reduced further or interrupted<br>– Study treatment should be dose interrupted if upper limits of systolic BP (≥ 160 mm Hg) are sustained and not adequately manageable or if systolic BP is > 180 mm Hg or diastolic BP > 110 mm Hg, or if subject is symptomatic<br>– Re-start study treatment at the most tolerable dose and re-escalate only if BP falls to and is sustained at < 150 mm Hg systolic and < 100 mm Hg diastolic

Hypertensive emergency[B]	– Permanently discontinue cabozantinib and hold nivolumab. Resumption of nivolumab monotherapy may be considered, after consultation with the trial PI, in patients who are deriving benefit and have fully recovered from this event.
BP, blood pressure.<br>[A]The investigator may decide to initiate or adjust antihypertensive treatment at a lower threshold than systolic BP >150 or diastolic BP >100 based on their clinical judgment and assessment of the individual subject.<br>[B] Hypertensive emergency is defined as uncontrolled elevated blood pressure with clinical evidence of progressive or impending end-organ damage (eg, myocardial infarction/ischemia, intracranial hemorrhage, cerebral ischemia, pulmonary edema, encephalopathy, kidney damage).	

Osteonecrosis of the Jaw

Osteonecrosis of the jaw (ONJ) has been reported in subjects treated with cabozantinib. Additional risk factors for ONJ have been identified including the use of bisphosphonates and denosumab, chemotherapy, corticosteroids, local radiotherapy, and dental or orofacial surgery procedures.
Osteonecrosis of the jaw can manifest as jaw pain, osteomyelitis, osteitis, bone erosion, tooth or periodontal infection, toothache, gingival ulceration, or gingival erosion. Persistent pain or slow healing of the mouth or jaw after dental surgery may also be manifestations of ONJ.
Perform an oral examination prior to initiation of cabozantinib and periodically during cabozantinib therapy. Advise subjects regarding oral hygiene practice and to quickly report symptoms to investigator. Caution should be used in subjects receiving bisphosphonates.
Invasive dental procedures should be avoided. In cases where dental procedures are unavoidable, treatment with cabozantinib should be held for at least 4 weeks prior to the procedure and resumed after complete wound healing has occurred. Bone healing may often require a protracted time.

Nausea and Vomiting

Nausea and vomiting has been reported among subjects treated with cabozantinib. Antiemetic agents are recommended as clinically appropriate at the first sign of nausea and vomiting or as prophylaxis to prevent emesis, along with supportive care in accordance to clinical practice guidelines. The 5-HT3 receptor antagonists are recommended over chronic use of NK-1 receptor antagonists and dexamethasone (NK-1 receptor antagonists can induce or inhibit CYP3A4, and glucocorticoids induce CYP3A4 and thus could lower cabozantinib exposure. Caution is also recommended with the use of nabilone, which is a weak inhibitor of CYP3A4. When therapy with antiemetic agents does not control the nausea or vomiting to tolerable levels, study treatment should be temporarily interrupted or dose reduced.
Dehydration may be associated with vomiting and monitoring for and correction of fluid and electrolyte disturbances should be implemented.

Neurological adverse events

Grade 1	– Continue nivolumab and cabozantinib.
Grade 2	– Hold nivolumab and cabozantinib.<br>Treat symptoms per local guidelines<br>– Consider 0.5-1 mg/kg/day methylprednisolone IV or PO equivalent.
Grade 3-4	– Permanently discontinue cabozantinib and nivolumab.<br>– Obtain neurology consult.<br>– Treat symptoms per local guidelines. Consider 1.0-2.0 mg/kg/day IV methylprednisolone IV or IV equivalent. Add prophylactic antibiotics for opportunistic infections.

Palmar-plantar erythrodysesthesia syndrome (PPES; also known as hand-foot syndrome)

PPES has been reported among subjects treated with cabozantinib. All subjects on study should be advised regarding avoidance of exposure of hands and feet to hot water, removal of calluses, protection of pressure-sensitive areas of hands and feet, and use of thick cotton gloves and socks to prevent injury and to keep the palms and soles dry. Early signs of hand-foot syndrome could be tingling, numbness, and slight redness or mild hyperkeratosis. Early manifestations include painful, symmetrical red and swollen areas on the palms and soles. The lateral sides of the fingers or peri-ungual zones may also be affected. Adequate interventions are required to prevent worsening of skin symptoms such as blisters, desquamations, ulcerations, or necrosis of affected areas. Aggressive management of symptoms is recommended, including early dermatology referral. In the case of study treatment-related skin changes (eg, rash, hand-foot syndrome), the investigator may request that additional assessments be conducted with the subject's consent. These assessments may include digital photographs of the skin changes and/or a biopsy of the affected skin and may be repeated until the skin changes resolve.

Grade 1	– Cabozantinib and nivolumab may be continued at the current dose if PPES is clinically insignificant and tolerable. Otherwise, cabozantinib should be reduced to the next lower dose level. Start urea 20% cream twice daily AND clobetasol 0.05% cream once daily. Reassess at least weekly; if PPES worsens at any time or does not improve after 2 weeks, proceed to the intervention guidelines for Grade 2.
Grade 2	– Cabozantinib and nivolumab may be continued if PPES is tolerated. Cabozantinib should be dose reduced or interrupted if PPES is intolerable; nivolumab can be continued as monotherapy. Continue urea 20% cream twice daily AND clobetasol 0.05% cream once daily and add analgesics (eg, NSAIDs/gamma-aminobutyric acid agonists) for pain control if needed. Reassess at least weekly; if PPES worsens or affects self-care, proceed to the intervention guidelines for Grade 3.
Grade 3	– Continue nivolumab but hold cabozantinib until severity decreases to Grade 1 or 0. Continue treatment of skin reaction with clobetasol 0.05% cream twice daily AND analgesics. Resume cabozantinib at one dose reduction if PPES recovers to Grade ≤ 1. Permanently discontinue cabozantinib if PPES does not improve within 4 weeks. Patients may continue nivolumab monotherapy.

Pulmonary adverse events

Grade 1 (radiographic changes only)	– Continue nivolumab and cabozantinib, but monitor closely for symptoms. Reimage at least every 3 weeks. If imaging worsens, treat as grade 2-4.
Grade 2	– Hold nivolumab and cabozantinib. Obtain pulmonary and ID consults.<br>– Monitor symptoms daily, and consider hospitalization.<br>– 1 mg/kg/day methylprednisolone IV or oral equivalent.<br>– Consider bronchoscopy and lung biopsy.<br>– If not improving after 2 weeks or worsening, treat as grade 3-4.
Grade 3-4	– Permanently discontinue nivolumab and cabozantinib. Obtain pulmonary and ID consults.<br>– Hospitalize; consider bronchoscopy and lung biopsy.<br>– 2-4 mg/kg/day methylprednisolone IV or IV equivalent, and add prophylactic antibiotics for opportunistic infections. Consider additional immunosuppression if not improving after 48 hours or worsening. Taper steroids over at least 6 weeks if improves to baseline.

Proteinuria

Proteinuria is an anticipated AE with the inhibition of VEGF pathways and has been reported among subjects treated with cabozantinib, and nephrotic syndrome has been reported with cabozantinib and other inhibitors of VEGF pathways. Cabozantinib should be permanently discontinued in subjects who develop nephrotic syndrome (proteinuria > 3.5 grams per day in combination with hypoalbuminemia and peripheral edema [hyperlipidemia and thrombotic disease may also be present]) or any other relevant renal disease.

Severity of Proteinuria (UPCR)	– Management of Proteinuria
≤ 1 mg/mg (≤ 113.1 mg/mmol)	– No change in cabozantinib treatment or monitoring
> 1 and < 3.5 mg/mg (> 113.1 and < 395.9 mg/mmol)	– Consider confirming with a 24-hour protein assessment within 7 days<br>– No change in cabozantinib treatment required if UPCR ≤ 2 mg/mg or urine protein ≤ 2 g/24 hours on 24-hour urine collection.<br>– Dose reduce or interrupt cabozantinib treatment if UPCR > 2 mg/mg on repeat UPCR testing or urine protein > 2 g/24 hours on 24-hour urine collection. Continue cabozantinib on a reduced dose if UPCR decreases to < 2 mg/mg. Consider holding cabozantinib treatment if UPCR remains > 2 mg/mg despite a dose reduction until UPCR decreases to < 2 mg/mg. Restart cabozantinib treatment at a reduced dose after a dose hold unless otherwise approved by sponsor.<br>– Repeat UPCR within 7 days and once per week. If UPCR < 1 mg/mg on 2 consecutive readings, UPCR monitoring can revert to protocol-specific times. (Second reading is confirmatory and can be done within 1 week of first reading.) If UPCR remains > 1 mg/mg and < 2 mg/mg for 1 month or is determined to be stable (< 20% change) for 1 month, check urine protein/creatinine per protocol or as clinically indicated.

≥ 3.5 mg/mg (≥ 395.9 mg/mmol)	– Hold cabozantinib treatment pending repeat UPCR within 7 days and/or 24-hour urine protein.<br>– If ≥ 3.5 mg/mg on repeat UPCR, continue to hold cabozantinib treatment and check UPCR every 7 days. If UPCR decreases to < 2 mg/mg, restart cabozantinib treatment at a reduced dose and monitoring of urine protein/creatinine should continue weekly until the UPCR decreases to < 1 mg/mg. If UPCR remains > 1 mg/mg and < 2 mg/mg for 1 month or is determined to be stable (< 20% change) for 1 month, check urine protein/creatinine per protocol or as clinically indicated.
Nephrotic syndrome	– Permanently discontinue cabozantinib and nivolumab
UPCR, urine protein/creatinine ratio.	

Renal

Grade 1	– Continue nivolumab and cabozantinib. Monitor creatinine weekly.
Grade 2-3	– Hold nivolumab and cabozantinib. Monitor creatinine every 2-3 days<br>– Consider treatment with 0.5-1.0 mg/kg/day methylprednisolone IV or oral equivalent.<br>– Consider renal biopsy with nephrology consult.<br>– Consider restarting nivolumab at fixed dose and cabozantinib at 1 dose reduction if creatinine resolves to ≤ grade 1, and systemic steroid dose is < 10 mg oral prednisone equivalent per day.
Grade 4	– Permanently discontinue nivolumab and cabozantinib. Obtain nephrology consult.<br>– Consider renal biopsy<br>– 1.0-2.0 mg/kg/day methylprednisolone IV or IV equivalent<br>– Taper steroids over at least 1 month and add prophylactic antibiotics for opportunistic infections.

Stomatitis and Mucositis

Stomatitis and mucositis has been reported among subjects treated with cabozantinib. Preventive measures may include a comprehensive oral examination to identify and treat any potential risk for complications before study treatment is initiated. Appropriate correction of local factors should be instituted as indicated, such as modification of ill-fitting dentures and appropriate care of gingivitis.

During treatment with cabozantinib good oral hygiene and standard local treatments such as nontraumatic cleansing and oral rinses (eg, with a weak solution of salt and baking soda) should be maintained. The oral cavity should be rinsed after meals, and dentures should be cleaned and brushed often to remove plaque. Local treatment should be instituted at the earliest onset of symptoms. Obtain bacterial/viral culture if oral infection is suspected and treat infection as indicated by local guidelines. When stomatitis interferes with adequate nutrition and local therapy is not adequately effective, dose reduction or temporary withholding of cabozantinib should be considered.

Thromboembolic Events

Deep vein thrombosis and PE has been reported among subjects treated with cabozantinib.

Subjects who develop a PE or DVT should have cabozantinib treatment held until therapeutic anticoagulation with heparins (eg, low molecular weight heparin [LMWH]) is established. Low molecular weight heparins are the preferred management for thrombotic events; oral anticoagulants (eg, warfarin or other coumarin-related agents, direct thrombin or direct FXa inhibitors, or antiplatelet agents, or chronic use of aspirin above low dose levels for cardioprotection per local applicable guidelines) are not allowed. Cabozantinib treatment may be resumed in subjects who are stable and have uncomplicated PE or DVT and are deriving clinical benefit from cabozantinib treatment and that anticoagulation does not place them at a significant risk that outweighs the benefit of resuming treatment per discretion of the investigator/Sponsor. During anticoagulation treatment, subjects need to be monitored on an ongoing basis for bleeding risk and signs of bleeding which may require additional or more frequent laboratory tests in accordance to institutional guidelines. If there are any signs of clinically significant bleedings, cabozantinib treatment should be permanently discontinued. Arterial thrombotic events (eg, transient ischemic attack, myocardial infarction) have been observed in studies with cabozantinib. Subjects should be evaluated for preexisting risk factors for arterial thrombotic events such as diabetes mellitus, hyperlipidemia, hypertension, coronary artery disease, history of tobacco use, and cardiac or thromboembolic events that occurred before initiation of study treatment. Cabozantinib treatment should be discontinued in subjects who develop an acute myocardial infarction, cerebral infarction or any other clinically relevant arterial thromboembolic complication.

QTc Prolongation

QTc prolongation has been reported among subjects treated with cabozantinib. If at any time on study there is an increase in QTc to an absolute value > 500 ms or an increase of > 60 ms above baseline, two additional ECGs must be performed with intervals not less than 3 min apart within 30 min after the initial ECG. If the average QTcF from the three ECGs is > 500 ms or increased by > 60 ms above baseline, the following actions should be taken:

- Withhold cabozantinib (nivolumab monotherapy may be continued)
- Hospitalize symptomatic subjects (eg, with palpitations, dizziness, syncope, orthostatic hypotension, a significant ventricular arrhythmia on ECG) for a thorough cardiology evaluation and management
- Consider cardiology consultation for asymptomatic subjects for evaluation and management
- Check electrolytes, especially magnesium, potassium and calcium; correct abnormalities as clinically indicated
- Check concomitant medications for any medication that may have contributed to QT prolongation, and if possible, discontinue these medications (http://www.qtdrugs.org)
- Repeat ECG triplicates hourly until the average QTcF is ≤ 500 msec, or otherwise determined by consultation with a cardiologist or appropriate expert
-

Subjects with QTc prolongation and symptoms should be monitored closely until the QTc elevation and symptoms have resolved. Nivolumab may be continued and cabozantinib may be restarted at a reduced dose level if all of the following conditions are met:

- Symptoms are determined to be unrelated to the QT interval prolongation
- The QTcF value > 500 ms or increase of > 60 ms above baseline is not confirmed according to protocol procedures

- Study treatment has been interrupted through a minimum of 1 week following the return of the QTcF to ≤ 500 msec or return to ≤ 60 ms above baseline.
- QT prolongation can be unequivocally associated with an event other than cabozantinib administration and is treatable/has been resolved

Following reinitiation of study treatment, ECGs must be repeated weekly for 2 weeks, then every 2 weeks for 1 month, then according to the protocol-defined time points.
Cabozantinib must be permanently discontinued if either of the following applies:

- Cardiac evaluation confirms that symptoms are the consequence of QT interval prolongation
- Recurrence of QTcF prolongation (confirmed by central ECG lab) after reinitiation of study treatment at a reduced dose

APPENDIX E SAE REPORTING FORM

<table>
<tr><td>Protocol Title:</td><td colspan="3">Feasibility and Efficacy of Neoadjuvant Cabozantinib plus Nivolumab (CaboNivo) Followed by Definitive Resection for Patients with Locally Advanced Hepatocellular Carcinoma (HCC)</td></tr>
<tr><td>Protocol Number:
J17136</td><td>Signature of PI:</td><td>Principal Investigator:</td><td>Date:</td></tr>
<tr><td>Report Type:
☐Initial
☐Follow-up
☐Final Follow-up
☐Death
☐Addendum to:</td><td>Serious Criteria (check all that apply):
☐Death
☐Life-threatening
☐ Hospitalization or Elongation of Existing Hospitalization
☐ Persistent or Significant Disability
☐ Congenital Anomaly
☐ Other Important Medical Event
☐ Cancer
☐ Overdose</td><td>Hospital Admission Date:

Hospital Discharge Date:</td><td>Date Event Discovered:</td></tr>
</table>

<table>
<tr><td colspan="3">Section A: Subject Information</td></tr>
<tr><td>Subject ID:</td><td>Subject Initial:</td><td>Subject Gender:
☐Male
☐Female</td></tr>
</table>

<table>
<tr><td colspan="3">Section B: Event Information</td></tr>
<tr><td rowspan="3">Event diagnosis or symptoms:</td><td>Date of First Dose:</td><td rowspan="3">Action taken with the study drug:
☐None
☐Interrupted
☐Discontinued
☐Delayed</td></tr>
<tr><td>Date of Last Dose Prior to Event:</td></tr>
<tr><td>Number of Total Doses:</td></tr>
</table>

**Event Onset Date:**	**Event End Date:**

**Relationship to:**	**Nivolumab**	**Cabozantinib**	**Underlying Disease**
**Unrelated**	☐	☐	☐
**Related**	☐	☐	☐

**Section C: Brief Description of the Event:**					
**Section D: Relevant Medical History**					
**Section E: Concomitant Drug (Not related to SAE)**					
**Name of the Drug**	**Start Date**	**Stop Date**	**Route**	**Dose**	**Frequency**
**Section F: Comments**					
**Additional Documents:** ☐ Please specify					

APPENDIX F RESPONSE EVALUATION CRITERIA IN SOLID TUMORS (RECIST) 1.1 CRITERIA FOR EVALUATING RESPONSE IN SOLID TUMORS

RECIST version 1.1 will be used in this study for assessment of tumor response. Either CT or MRI may be used utilized, as per RECIST 1.1.

Disease Parameters

Measurable disease: Measurable lesions are defined as those that can be accurately measured in at least one dimension (longest diameter to be recorded) as ≥20 mm by chest x-ray, as ≥10 mm with CT scan, or ≥10 mm with calipers by clinical exam. All tumor measurements must be recorded in millimeters (or decimal fractions of centimeters).

Non-measurable disease: All other lesions (or sites of disease), including small lesions (longest diameter <10 mm or pathological lymph nodes with ≥10 to <15 mm short axis), are considered non-measurable disease. Bone lesions, leptomeningeal disease, ascites, pleural/pericardial effusions, lymphangitis cutis/pulmonitis, inflammatory breast disease, and abdominal masses (not followed by CT or MRI), are considered as non-measurable.

> Note: Cystic lesions that meet the criteria for radiographically defined simple cysts should not be considered as malignant lesions (neither measurable nor non-measurable) since they are, by definition, simple cysts.
>
> 'Cystic lesions' thought to represent cystic metastases can be considered as measurable lesions, if they meet the definition of measurability described above. However, if non-cystic lesions are present in the same patient, these are preferred for selection as target lesions.

Target lesions: All measurable lesions up to a maximum of 2 lesions per organ and 5 lesions in total, representative of all involved organs, should be identified as **target lesions** and recorded and measured at baseline. Target lesions should be selected on the basis of their size (lesions with the longest diameter), be representative of all involved organs, but in addition should be those that lend themselves to reproducible repeated measurements. It may be the case that, on occasion, the largest lesion does not lend itself to reproducible measurement in which circumstance the next largest lesion which can be measured reproducibly should be selected. A sum of the diameters (longest for non-nodal lesions, short axis for nodal lesions) for all target lesions will be calculated and reported as the baseline sum diameters. If lymph nodes are to be included in the sum, then only the short axis is added into the sum. The baseline sum diameters will be used as reference to further characterize any objective tumor regression in the measurable dimension of the disease.

Non-target lesions: All other lesions (or sites of disease) including any measurable lesions over and above the 5 target lesions should be identified as **non-target lesions** and should also be recorded at baseline. Measurements of these lesions are not required, but the presence, absence, or in rare cases unequivocal progression of each should be noted throughout follow-up.

Evaluation of Target Lesions

Complete Response (CR): Disappearance of all target lesions. Any pathological lymph nodes

(whether target or non-target) must have reduction in short axis to <10 mm.

Partial Response (PR): At least a 30% decrease in the sum of the diameters of target lesions, taking as reference the baseline sum diameters.

Progressive Disease (PD): At least a 20% increase in the sum of the diameters of target lesions, taking as reference the smallest sum on study (this includes the baseline sum if that is the smallest on study). In addition to the relative increase of 20%, the sum must also demonstrate an absolute increase of at least 5 mm. (Note: the appearance of one or more new lesions is also considered progressions).

Stable Disease (SD): Neither sufficient shrinkage to qualify for PR nor sufficient increase to qualify for PD, taking as reference the smallest sum diameters while on study.

Evaluation of Non-Target Lesions

Complete Response (CR): Disappearance of all non-target lesions. All lymph nodes must be non-pathological in size (<10 mm short axis).

Non-CR/Non-PD: Persistence of one or more non-target lesion(s) and/or maintenance of tumor marker level above the normal limits.

Progressive Disease (PD): Appearance of one or more new lesions and/or *unequivocal progression* of existing non-target lesions. *Unequivocal progression* should not normally trump target lesion status. It must be representative of overall disease status change, not a single lesion increase.

Although a clear progression of "non-target" lesions only is exceptional, the opinion of the treating physician should prevail in such circumstances, and the progression status should be confirmed at a later time by the review panel (or Principal Investigator).

Evaluation of Best Overall Response

The best overall response is the best response recorded from the start of the treatment until disease progression/recurrence (taking as reference for progressive disease the smallest measurements recorded since the treatment started). The patient's best response assignment will depend on the achievement of both measurement and confirmation criteria.

For Patients with Measurable Disease (i.e., Target Disease)

Target Lesions	Non-Target Lesions	New Lesions	Overall Response	Best Overall Response when Confirmation is Required*
CR	CR	No	CR	≥4 wks. Confirmation**
CR	Non-CR/Non-PD	No	PR	≥4 wks. Confirmation**
CR	Not evaluated	No	PR	
PR	Non-CR/Non-PD/not evaluated	No	PR	
SD	Non-CR/Non-PD/not evaluated	No	SD	Documented at least once ≥4 wks. from baseline**
PD	Any	Yes or No	PD	no prior SD, PR or CR
Any	PD***	Yes or No	PD	
Any	Any	Yes	PD	

* See RECIST 1.1 manuscript for further details on what is evidence of a new lesion.

** Only for non-randomized trials with response as primary endpoint.

*** In exceptional circumstances, unequivocal progression in non-target lesions may be accepted as disease progression.

<u>Note</u>: Patients with a global deterioration of health status requiring discontinuation of treatment without objective evidence of disease progression at that time should be reported as "*symptomatic deterioration.*" Every effort should be made to document the objective progression even after discontinuation of treatment.

<u>Reference</u>

E.A. Eisenhauer, P. Therasse, J. Bogaerts, L.H. Schwartz, D. Sargent, R. Ford, J. Dancey, S. Arbuck, S.

Gwyther, M. Mooney, L. Rubinstein, L. Shankar, L. Dodd, R. Kaplan, D. Lacombe, J. Verweij. New response evaluation criteria in solid tumours: Revised RECIST guideline (version 1.1). Eur J Cancer. 2009 Jan;45(2):228-47.

APPENDIX G IMMUNE RELATED RESPONSE CRITERIA (IRRC) CRITERIA FOR EVALUATING RESPONSE IN SOLID TUMORS

Definitions of measurable and non-measurable disease

Measurable disease: Neoplastic masses that can be precisely measured in 2 in-plane perpendicular diameters. Both its longest diameter and its longest perpendicular must be greater than or equal to 10 mm. Lymph nodes must have a short-axis line-length of ≥ 15 mm. Malignant lymph nodes must be measurable in 2 perpendicular diameters. Both its longest diameter and its longest perpendicular must be greater than or equal to 15 mm. The quantitative endpoint will be defined as the product of the longest diameter with its longest perpendicular.

Non-measurable disease: Non-measurable lesions are those that are not suitable for quantitative assessment over time. These include:

1) Neoplastic masses that are too small to measure, because their longest uninterrupted diameter or longest perpendicular are less than 10 mm.

2) Neoplastic masses whose boundaries cannot be distinguished. This includes masses which cannot be demarcated from surrounding tissue because of inadequate contrast, masses with overly complex morphology, or those with highly heterogeneous tissue composition.

3) Other types of lesions that are confidently felt to represent neoplastic tissue, but difficult to quantify in a reproducible manner. These include bone metastases, leptomeningeal metastases, malignant ascites, pleural/pericardial effusions, inflammatory breast disease, lymphangitis cutis/pulmonis, cystic lesions, ill-defined abdominal masses, etc.

For irRC, only target lesions selected at baseline and measurable new lesions are taken into account.

At the baseline tumor assessment, the sum of the products of the two largest perpendicular diameters (SPD) of all **index lesions** (five lesions per organ, up to 10 visceral lesions and five cutaneous index lesions) is calculated.

At each subsequent tumor assessment, the SPD of the index lesions and of new, measurable lesions (≥ 5 X 5 mm; up to 5 new lesions per organ: 5 new cutaneous lesions and 10 visceral lesions) are added together to provide the total time-point **tumor burden.**

Overall response using irRC:

- **Complete Response (irCR):** Complete disappearance of all tumor lesions (whether measureable or not, and no new lesions). CR must be confirmed by repeated, consecutive assessments made no less than 4 weeks from the date first documented.

- **Partial Response (irPR):** Decrease in SPD of 50% or greater by a consecutive assessment at least 4 weeks after first documentation.

- **Stable Disease (irSD):** Failure to meet criteria for irCR or irPR, in absence of irPD.

- **Progressive Disease (irPD):** At least 25% increase in SPD relative to nadir (minimum recorded tumor burden) Confirmation by a repeat, consecutive assessment no less than 4 weeks from the data first documented.

Please note other key differences between irRC and the original WHO criteria:

New measurable lesions will be incorporated into the SPD

New non measurable lesions do not define progression but preclude irCR

Non-index lesions contribute to defining irCR (complete disappearance required).

See the Investigators Imaging Operations Manual (IIOM) for more details.

REFERENCE

IrRC for the current protocol is adopted from the following reference:

Wolchok, JD, Hoos, A, O'Day S, et al., Guidelines for the Evaluation of Immune Therapy Activity in Solid Tumors: Immune-Related Response Criteria. Clinical Cancer Research, 2009 Dec 1;15(23):7412-20. Epub 2009 Nov 24
